# Valorizing Assorted Logging Residues: Response Surface Methodology in the Extraction Optimization of a Green Norway Spruce Needle-Rich Fraction To Obtain Valuable Bioactive Compounds

**DOI:** 10.1021/acssusresmgt.3c00050

**Published:** 2024-02-02

**Authors:** Jenni Tienaho, Marina Fidelis, Hanna Brännström, Jarkko Hellström, Magnus Rudolfsson, Atanu Kumar Das, Jaana Liimatainen, Anuj Kumar, Mika Kurkilahti, Petri Kilpeläinen

**Affiliations:** † Production Systems, Natural Resources Institute Finland (Luke), Latokartanonkaari 9, FI-00790 Helsinki, Finland; ‡ Food Sciences Unit, Department of Life Technologies, University of Turku, FI-20014 Turku, Finland; § Production Systems, Natural Resources Institute Finland (Luke), Teknologiakatu 7, FI-67100 Kokkola, Finland; ∥ Production Systems, Natural Resources Institute Finland (Luke), Myllytie 1, FI-31600 Jokioinen, Finland; ⊥ Unit of Biomass Technology and Chemistry, Swedish University of Agricultural Sciences, SE-901 83 Umeå, Sweden; # Natural Resources, Natural Resources Institute Finland (Luke), Itäinen Pitkäkatu 4 A, FI-20520 Turku, Finland

**Keywords:** antibacterial, antioxidant, condensed tannins, extraction optimization, industrially assorted needle-rich logging residue, *Picea abies*, response surface methodology, total phenolic content

## Abstract

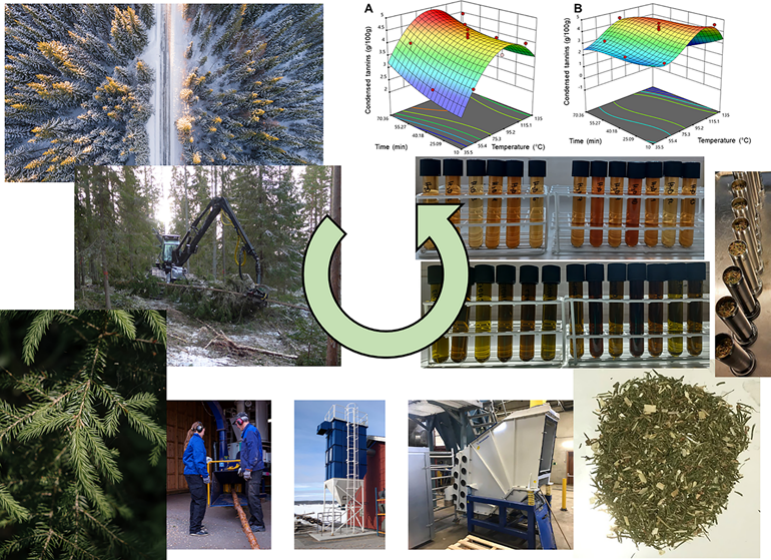

During stemwood harvesting, substantial volumes of logging residues are produced as a side stream. Nevertheless, industrially feasible processing methods supporting their use for other than energy generation purposes are scarce. Thus, the present study focuses on biorefinery processing, employing response surface methodology to optimize the pressurized extraction of industrially assorted needle-rich spruce logging residues with four solvents. Eighteen experimental points, including eight center point replicates, were used to optimize the extraction temperature (40–135 °C) and time (10–70 min). The extraction optimization for water, water with Na_2_CO_3_ + NaHSO_3_ addition, and aqueous ethanol was performed using yield, total dissolved solids (TDS), antioxidant activity (FRAP, ORAC), antibacterial properties (*E. coli*, *S. aureus*), total phenolic content (TPC), condensed tannin content, and degree of polymerization. For limonene, evaluated responses were yield, TDS, antioxidant activity (CUPRAC, DPPH), and TPC. Desirability surfaces were created using the responses showing a coefficient of determination (*R*^2^) > 0.7, statistical significance (*p* ≤ 0.05), precision > 4, and statistically insignificant lack-of-fit (*p* > 0.1). The optimal extraction conditions were 125 °C and 68 min for aqueous ethanol, 120 °C and 10 min for water, 111 °C and 49 min for water with Na_2_CO_3_ + NaHSO_3_ addition, and 134 °C and 41 min for limonene. The outcomes contribute insights to industrial logging residue utilization for value-added purposes.

## Abstract 2

Sustainability is obtained by optimizing industrially assorted fresh logging residue extraction with green solvents and mild extraction conditions to obtain bioactive and polyphenol-rich extracts.

## Introduction

Logging residues are defined as the above-ground biomass left to the felling sites after harvesting the stem wood material, including the tops and branches of harvested trees and small diameter trees from thinnings.^1^ High volumes of logging residues are produced yearly. In Finland alone, 4.392 million dry tons of spruce logging residues (branches and needles) are available annually.^2^ The share of Norway spruce needles has been estimated to cover 30% of the total crown biomass.^3^ Logging residues account for a considerable proportion of the total nutrient pool originally bound in the growing stand,^4^ e.g., nearly 80% of the total N and as much as 90% of the total P of the standing tree biomass pools of these nutrients. Forest litter plays an essential role in the formation of soil humus, which is crucial for soil fertility and nutrient cycling.^5^ Therefore, logging residues are mostly left at the sites to release the nutrients back to forest soils.^6,7^ However, to achieve the renewable energy targets (e.g., in the European Union, Directive 2009/28/EC), there has been a recent increase in the utilization of logging residues for forest-based bioenergy production,^8−10^ while the high cost of logging residue transportation and dry mass losses discourage bioenergy production from these types of biomasses.^11^ Besides nutrient recycling and bioenergy production, logging residues offer potential as alternate lignocellulosic materials for several higher value applications, such as reinforcement biomass for biocomposites^12^ and growing media.^13^ When separating valuables from logging residues before potential energy use or lignocellulosic applications, it is possible to increase the efficient use of natural resources and promote sustainable development. For example, logging residues could be directed to the extraction of valuable compounds, and the remaining material could be utilized as a source of other bioproducts, biochemicals, or bioenergy.^12^ These nature-derived ingredients open possibilities for replacing fossil-based products. However, most of this potential has not been utilized due to the high costs of harvesting, transport, storing, and handling.

Logging residues, especially needles, contain high amounts of valuable extractable compounds. Woody biomass and logging residue extractives can be classified into three groups: aliphatic compounds (e.g., terpenes, terpenoids, fatty acids, and resin acids), phenolic compounds (e.g., stilbenes, lignans, flavonoids, and tannins), and other compounds (e.g., sugars, amino acids, quinones, and alkaloids).^14^ Needles contain vitamins, bioactive extractives (up to 43% of dry matter), and protein (about 10% of dry matter).^15,16^ In fact, over 200 compounds have been identified from conifer sprouts and needles, and their chemistry has been found to differ from sap and heartwood compounds.^17−19^ Especially, secondary metabolite-related extractives provide defense for the standing trees against different abiotic and biotic stressors, such as excessive humidity, drought, temperature variation, parasites, bacteria, fungi, and other phytopathogenic organisms.^20^ Thus, it is common for woody biomass extractives to possess antioxidant and antimicrobial properties, making them useful as preservatives in the food and cosmetic industries and holding potential for medicinal products.^21^ Even without considering the phenomenon of increased antibiotic-resistant pathogens, current antibiotics often cause adverse effects and have difficulties in safe dosing for various individuals.^22^ Consequently, there is a demand and commercial opportunity for effective, safe, and environmentally friendly antibiotic and antimicrobial substances, and metabolites obtained from abundant woody biomass could offer an attractive source. At the same time, the global market for extractives and other biobased products is growing.^23^ Forest biomass-based extractives are potential raw materials to produce a range of added-value products, such as pharmaceuticals or cosmetic ingredients,^24^ platform and specialty chemicals, and dietary supplements.^25,26^ Forest biomass can also be converted into biopolymers,^27^ bioplastics,^28^ foams/emulsions, and coatings^29^ and used as a potential feedstock for liquid biofuels.^10^

Currently, no remarkable utilization of logging residues or needle-based extractive compounds exists in Finland or Sweden. Since there is no industrial utilization of this biomass assortment as a source of biochemicals, methods for its refining require development. Logging residues have a complex and varied nature, and the needles are rich in chemicals, such as waxes, which many biorefining processes cannot handle. Separation of the needles for the extraction of high-value chemicals can improve the quality of the remaining fraction, which can then be used by other processes.^12^ However, many existing studies rely on handpicking small quantities of pure needle biomasses, and limited information is available on samples obtained from the industrial-scale assorted fresh biomass material. In addition to the biomass assortment, extraction efficiency is influenced by parameters like the solvent composition, the extraction temperature and time, the particle size of the material to be extracted, the liquid-to-solid ratio, and the pH value.^30,31^ The properties of the extracted compounds need consideration to avoid unnecessary chemical modification during extraction by hydrolysis, oxidation, and isomerization reactions.^32^ Excessively high extraction temperatures could degrade targeted molecules, such as condensed tannins.^33^ Generally, extractions can be facilitated and higher yields obtained by increasing the temperature and solvent-to-solid ratio to favor solubilization and diffusion.^34,35^ However, excessively elevated temperatures not only cause the decomposition of thermolabile compounds but also lead to solvent losses and extracts containing impurities or unwanted compounds. Additionally, extraction efficiency increases only up to a certain point, and the extractable compounds of interest may begin to degrade when extraction time is prolonged.^36^ Pretreatment, conservation, and storage of plant material significantly influence extraction yield and must be carefully controlled. Generally, the extraction of plant material leads to the recovery of a wide variety of components.^31^ Thus, the obtained extract must undergo further treatment and refining before achieving the desired final form for different applications. Typically, the required treatments after extraction are (i) separation of solids, (ii) concentration of extracts via solvent evaporation, (iii) fractioning and enrichment of target components, (iv) removal of impurities, and (v) drying of the products.

Solvent properties, such as polarity, affect the composition of the biomass extract. In addition to physical solubility, extraction performance is directly related to solvent and solute similarities regarding functional groups. It is known that less polar solvents generally extract lower amounts of polyphenols. Usually, highly hydroxylated aglycone forms of polyphenols are soluble in water, alcohols (e.g., methanol, ethanol), or mixtures of these. In contrast, less polar and highly methoxylated aglycone forms are extracted through less polar solvents (e.g., acetone, ethyl acetate). Given that the hydroxyl groups of phenolic compounds contribute to the antioxidant activity, more polar extracts typically show higher antioxidant activities due to the rupture of cell membranes caused by the alcoholic solvent, providing endocellular extraction.^37,38^ It is also preferable to use solvents that are considered green based on their environmental, safety, and health effects.^39^ Water is an environmentally friendly polar solvent able to extract polar compounds, and water extraction conditions can be modified using chemical additions to adjust pH (e.g., Na_2_CO_3_^40^) and to react with condensed tannins (e.g., NaHSO_3_^41^) to enhance the extraction yield. Ethanol is a solvent able to extract both nonpolar (lipophilic) and polar (hydrophilic) compounds. In addition to water, ethanol is a common solvent approved by the European Union to extract food ingredients.^42^ Limonene (1-methyl-4-isopropenylcyclohex-1-ene) is a nonpolar monoterpene naturally found in Norway spruce (*Picea abies*) needles^43^ and woody materials.^44^ Among terpenes, limonene is a greener solvent alternative usually used for lipid extractions, which provides lower toxicity, environmental risk, and flammability than other conventional solvents, such as hexane.^45,46^ Limonene is also an edible food ingredient and used as a sweetener and fragrance in the food and cosmetics industries.^47^ Given its inherent presence in woody materials, limonene could be used in extractions, and the potential of recycling it back to the extraction process could further extend its life cycle in biorefinery applications.

This study aims to introduce environmentally friendly and industrially feasible processes for the utilization of extractives from logging residues. The logging residue branches were chipped, and the needle-rich fraction was separated with a cyclone followed by mechanical sieving. According to previous literature, the content of polyphenols, such as stilbenes, can be reduced by up to 40% after 10 h of drying.^48^ Logging residues also need to be collected and refined as fresh as possible to avoid the losses of extractives.^49,50^ Thus, in this study, the process was kept industrially feasible and rapid while avoiding energy-inefficient drying. Optimization of extraction time and temperature was performed against multiple responses, such as the extract bioactivities, which were detected using antioxidant and antibacterial analyses, and tannin quantification with chromatographic methods. Until now, the process optimizations for hydrothermal extraction of logging residues have rarely been reported. Considering the complex chemical compositions of the logging residues, a robust and controlled optimization is needed for their efficient valorization. Given that multiple process parameters impact the extraction process and properties of extracted compounds, the response surface methodology (RSM) combined with the design of experiments (DOE) was chosen for optimization. The RSM is an effective mathematical and statistical tool for evaluating the effect of independent variables and their interactions.^51−54^ In the literature, the RSM and DOE optimization have mainly been reported in the ultrasonic extraction of different biomasses, including tree leaves, for the recovery of bioactive compounds.^51,55^

In this study, the pressurized extraction of industrially assorted logging residues using four solvents with differing characteristics was optimized. Simultaneously, up to 11 responses or target variables were considered using the RSM. This approach maximizes the potential to obtain industrially feasible valuable extracts characterized by varying polarity with a high concentration of condensed tannins and total phenolics as well as antioxidant and antibacterial properties.

## Experimental Section

### Collection and Assortment of Raw Material

**Figure 1:**
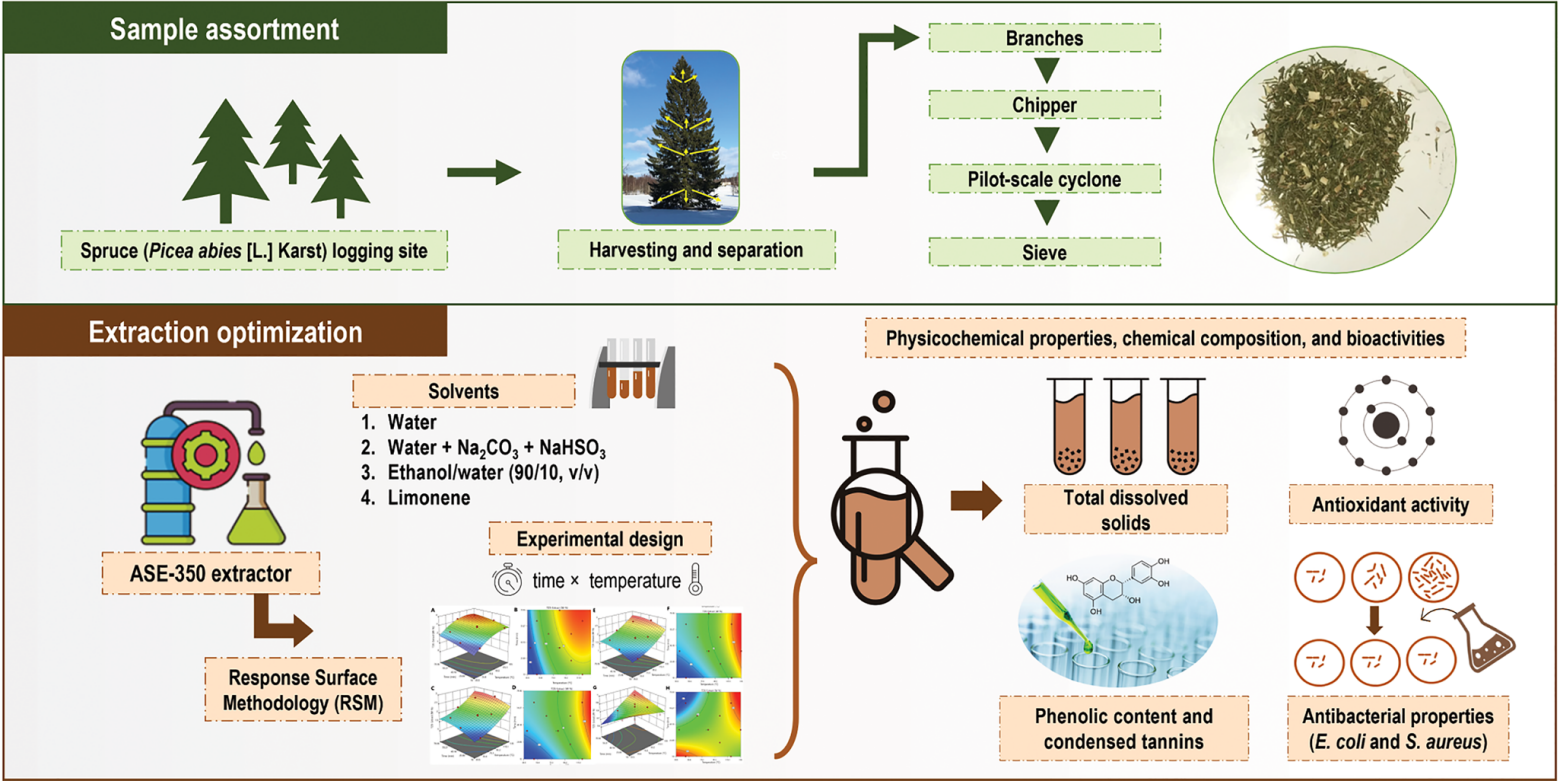
Overall study scheme.

The full study scheme of this investigation is presented in Figure 1. Logging residues used in the trials consisted of branches from a spruce (*Picea abies* [L.] Karst) harvested in Håknäs, Sweden (63°54′0″N and 19°74′1″E) in a 70-year-old stand. The harvesting and separation of green needles were completed within 1 week in May 2021. The separation work was carried out at the Biomass Technology Centre (BTC), Swedish University of Agricultural Sciences (SLU), Umeå, Sweden.

**Figure 2:**
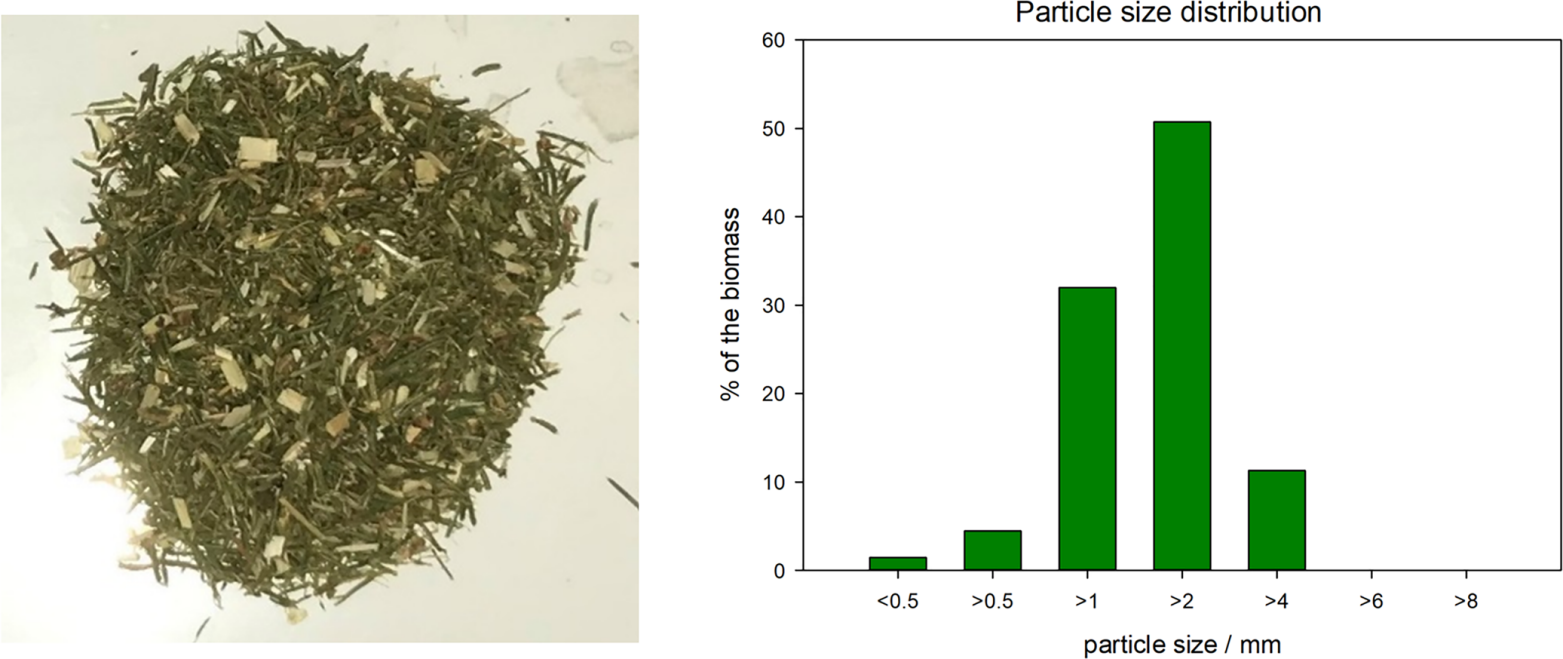
Needle-rich fraction obtained by the assortment procedure and the particle size distribution of the fraction.

The separation of green needles from the rest of the branch material occurred in three steps. Branches were chipped (Edsbyhuggen, Woxnadalens Energi AB, Sweden) to enable the feeding of the material to a pilot cyclone.^56^ The impact of the material fed through the cyclone allowed the separation of the needles from the rest of the branch material. This process facilitated the production of a fraction with a higher proportion of needles by mechanically sieving (Fredrik Mogensen AB, Mogensen Sizer E0554) the material, and the fraction with particle size ≤ 4 mm was used (Figure 2).

### Chemicals

(−)-Limonene (96%) was purchased from Acros Organics (Spain) and ethanol (99.5%) from Altia (Finland). Na_2_CO_3_ was from BDH (England) and NaHSO_3_ from Acros Organics (Belgium). If not otherwise mentioned, all other chemicals were obtained from Sigma-Aldrich (Sigma-Aldrich Chemie GmbH, Steinheim, Germany).

### Extraction Method and Solvents

Assorted logging residue samples were extracted with an accelerated solvent extractor (ASE-350, Dionex, USA) using four different solvents: water, water with Na_2_CO_3_ (20 g/L) and NaHSO_3_ (20 g/L), ethanol/water (90/10, v/v), and limonene. The amounts of fresh samples were adjusted according to the moisture content so that there would be 10 g of oven-dried sample in each extraction in a 100 mL extraction vessel. For Na_2_CO_3_ + NaHSO_3_ additions, the concentration of the extraction liquid was adjusted to ensure that, upon addition, the liquid and the moisture content in the fresh sample were combined, resulting in a total liquid concentration of 20 g/L for both Na_2_CO_3_ and NaHSO_3_ in the vessel. After the extractions, extracts were collected and stored in a freezer (−20 °C) before further analyses.

### Responses

The extraction optimization for water, water with Na_2_CO_3_ + NaHSO_3_ addition, and aqueous ethanol was performed using yield, total dissolved solids (TDS), antioxidant activity (FRAP, ORAC), antibacterial properties (*E. coli*, *S. aureus*), total phenolic content (TPC), condensed tannin content, and degree of polymerization. For limonene, evaluated responses were yield, TDS, antioxidant activity (CUPRAC, DPPH), and TPC. The detailed method descriptions for the individual responses can be found in the Supporting Information (Supplementary Document 1).

### Experimental Design and Statistical Optimization

The experimental design was created using the Design Expert DX13 V. 13.0.8.0 (StatEase, Minneapolis, USA) program. The response surface methodology (RSM) was employed to investigate the effects of time and temperature (factors) on the yield and TDS of the extracts, TPC, antioxidant capacities, antibacterial effects, and condensed tannins (responses). A central composite response surface design was utilized for each solvent with 18 runs, and the time and temperature combinations were chosen by Design Expert. The combinations were run in a randomized order. Analysis results were utilized to identify optimized conditions for forest residue extractions with each solvent. Optimization was performed using the Design Expert desirability function.^57^ The best model was selected among the first-, second-, or third-order polynomial models. Mathematical modeling, information on optimized factors, and the two-factor central composite quadratic design used for optimization can be found in the Supporting Information (Supplementary Document 1).

## Results and Discussion

In this section, results are primarily presented using the response surface models (see details in Supplementary Tables 1–4). All extraction run responses with each solvent can be found in the Supporting Information (Supplementary Table 5).

### Extraction Yield

The extraction yields (in mg/g original dry sample) are presented in Table 1, and the TDS RSMs are illustrated in the Supporting Information (Supplementary Figure 1**)**.

**Table 1:** Extraction Yields (mg/g original biomass DW) for All Solvents

			yield per original dry sample (mg/g)			
run	temperature (°C)	time (min)	aqueous ethanol	water	water with Na_2_CO_3_ + NaHSO_3_	limonene
1	120	60	245	194	207	65
2	85	35	196	147	142	54
3	40	35	113	116	85	48
4	50	60	147	131	102	50
5	85	35	196	142	145	58
6	50	10	107	97	69	51
7	85	70	211	153	159	64
8	135	35	221	218	203	63
9	85	35	194	140	139	63
10	120	10	197	159	158	57
11	85	35	188	142	140	55
12	85	35	185	147	139	64
13	103	48	207	165	175	58
14	85	35	187	145	137	62
15	85	35	191	147	142	57
16	68	48	170	130	127	48
17	103	23	194	154	170	62
18	85	35	186	138	155	64

Extraction yields increased as extraction time and temperature increased in all solvents except limonene (Table 1). The highest yields were obtained with aqueous (aq.) ethanol, followed by water and water with chemical additions. Solvents were selected to include water as a polar solvent for hydrophilic compounds, aqueous ethanol as a general solvent for both lipophilic and hydrophilic compounds, and a nonpolar solvent, limonene, for lipophilic compounds to cover the whole polarity range. Bioactive extractables from needles with varying polarities can belong to diverse chemical groups, such as terpenes, fatty acids, sterols, waxes, and phenolic compounds.^17−19^ However, the compound profiles can vary according to the maturity of the needles,^58^ moisture and nutrient availability from the soil,^59^ and solar UV radiation as well as seasonal differences.^60^ The overall extraction yield of water (97–218 mg/g) was like spruce bark hot water extraction yields (37–209 mg/g) obtained in previous literature.^40,61−64^ Aqueous ethanol extractions had a 107–245 mg/g yield range, while water with Na_2_CO_3_ and NaHSO_3_ additions had a slightly lower 69–207 mg/g yield range. Limonene results were contradictory due to the large variation of TDS results in the center point (Supplementary Figure 1). During oven drying for TDS determination, limonene extracts formed a hardened surface layer preventing evaporation. While this phenomenon was addressed using sand to break the surface tension, it is possible that this affected both the yield and the TDS for limonene extracts and resulted in larger replicate variation. Limonene yields were also lower than with other solvents as it can mainly extract nonpolar compounds. Lack of fit *p* values for the repeated extraction conditions in the middle were all not significant, which indicates that the models fitted rather well: 0.5018, 0.6273, 0.6273, and 0.5962 for aq. ethanol, water, water with Na_2_CO_3_ + NaHSO_3_, and limonene, respectively.

### Total Phenolics

The phenolic content increased as the extraction temperature and time increased (Supplementary Figure 2). Overall, the results are in the same range as those in previous studies published using similar extraction parameters (e.g., Pap et al.^64^ for spruce bark). In the models for aq. ethanol, water, and limonene, large variation was observed in the middle point replicated 8 times. The variation was lower for water with Na_2_CO_3_ + NaHSO_3_ addition extracts, where TPC values were mostly in the same range as those in aq. ethanol and water extracts, but temperatures over 100 °C increased the TPC values up to 23.81 mg GAE/g. It can be speculated that this increase is caused by the degradation of lignin polymers, which includes phenolic compounds that could then be detected by this method.^65,66^ The TPC values were 10-fold higher for the limonene extracts. Instead of the Prussian blue methodology used for other extractions, the modified Folin–Ciocalteu method used for limonene extracts enables simultaneous measurement of lipophilic and hydrophilic polyphenols. This indicates that the Folin–Ciocalteu test method is more sensitive to the phenolics from coniferous extracts, and this hypothesis is also supported by previous literature.^67^

### Antioxidant Properties

**Figure 3:**
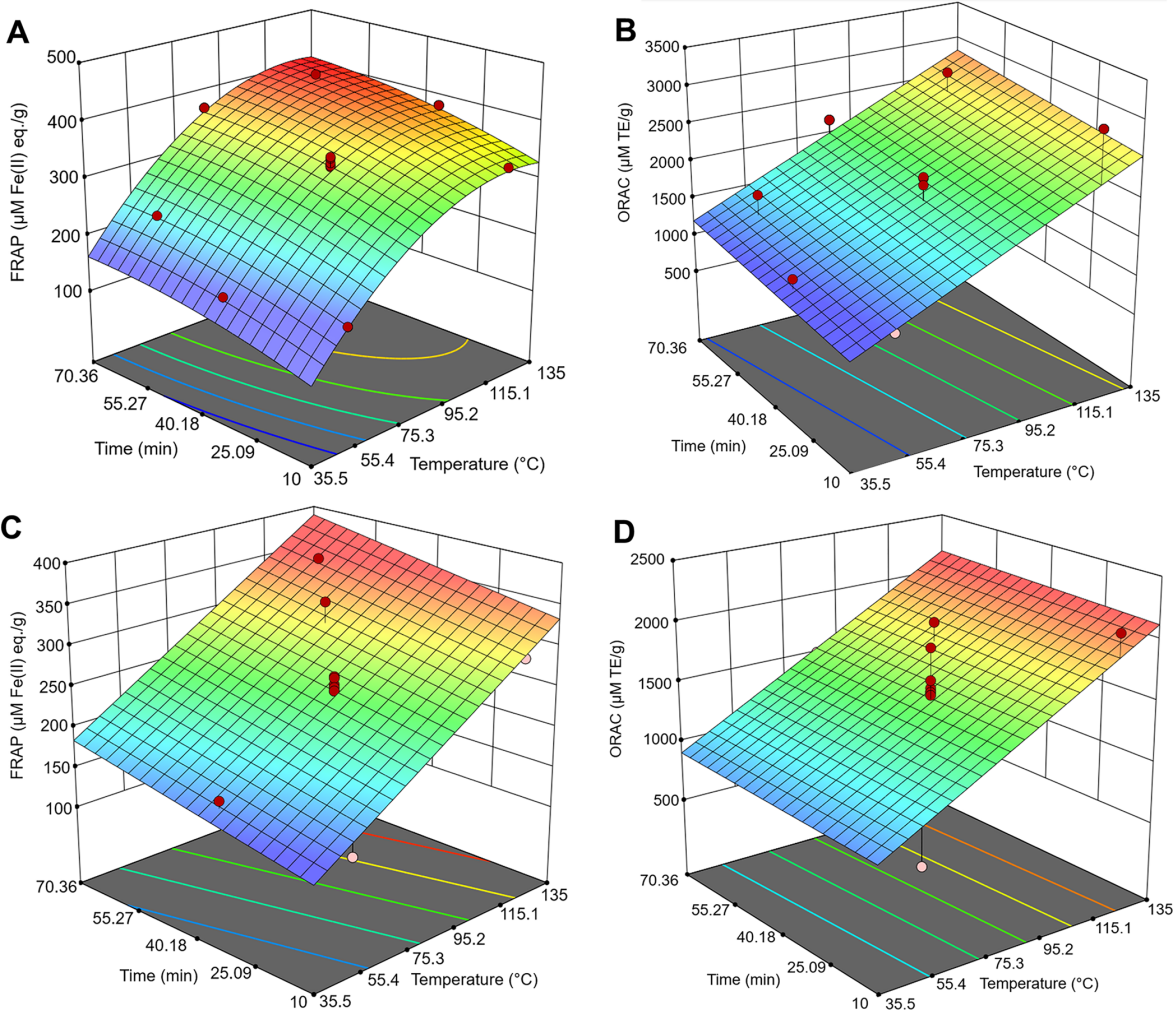
Antioxidant test result response surfaces for aq. ethanol extracts (A) FRAP (μM Fe(II) eq/g) and (B) ORAC (μM TE/g) and water extracts (C) FRAP and (D) ORAC. RSM was quadratic for A (*R*^2^ = 0.9687) and linear for B (*R*^2^ = 0.9234), C (*R*^2^ = 0.7127), and D (*R*^2^ = 0.7154).

The obtained FRAP values were max 355 μM Fe(II) eq/g for water and 411 μM Fe(II) eq/g for aq. ethanol extraction. Jyske et al.^15^ found that freshly frozen needle biomass extractions yielded FRAP values of approximately 800 μM Fe(II) eq/g for both water extraction and 70:30 (vol %) ethanol/water extraction. Our results are lower, which can result from the industrially feasible assortment not producing a completely pure needle fraction for the extraction. However, ORAC values obtained in this study are within the same range as those found by Jyske et al.,^15^ suggesting that ORAC active substances are not as susceptible to changes in the sampling, assortment, and handling procedures. From the antioxidant response surface models of aq. ethanol and water extracts (Figure 3), the variation in the center point was low in all but ORAC for water extracts (Figure 3D). All response surface models showed a high enough coefficient of determination values (*R*^2^) to be considered for the optimization. For aq. ethanol extraction, the highest FRAP values were obtained with 120 °C and 60 min extraction, and in temperatures over that (135 °C), the antioxidant results were lower. In contrast, with ORAC for aq. ethanol extracts and both FRAP and ORAC for water extracts, the values increased in a linear manner as the extraction temperature and time increased.

Water with Na_2_CO_3_ + NaHSO_3_ and limonene extracts showed more variation both in the center point and also from the response surface models (Supplementary Figure 3). The best-fit models were chosen, and only FRAP for water with Na_2_CO_3_ + NaHSO_3_ extracts and DPPH for limonene extracts showed high enough coefficient of determination values (*R*^2^ > 0.7) to be considered for optimization. In all but DPPH for limonene extracts, the higher the extraction temperature and time, the higher the expected values, whereas the expected DPPH results seemed to be more dependent on the temperature than time. DPPH values of the obtained lipid fractions in this study were low when compared to previous literature (e.g., Pap et al.^64^ for spruce bark extraction with water), and the differences between runs with different extraction parameters were small. Limonene is a nonpolar solvent, and the polarity and water solubility of extractables have been found to affect the antioxidant activities. As an example, Hofmann et al. found that catechins and their oligomers (procyanidins) had the highest levels in the 10–20% v/v acetone extracts, flavonoid glycosides were best soluble in 30–50% v/v acetone solutions, while derivatives of phenolic acids and stilbenes had the highest levels in 50–60% v/v acetone extracts.^68^ In addition, limonene itself has been found to possess antioxidant properties in the DPPH assay.^69^ Thus, while the expected activities were lower than those with polar solvents, the inherent solvent activity can also mask small differences in results.

### Antibacterial Analyses

**Figure 4:**
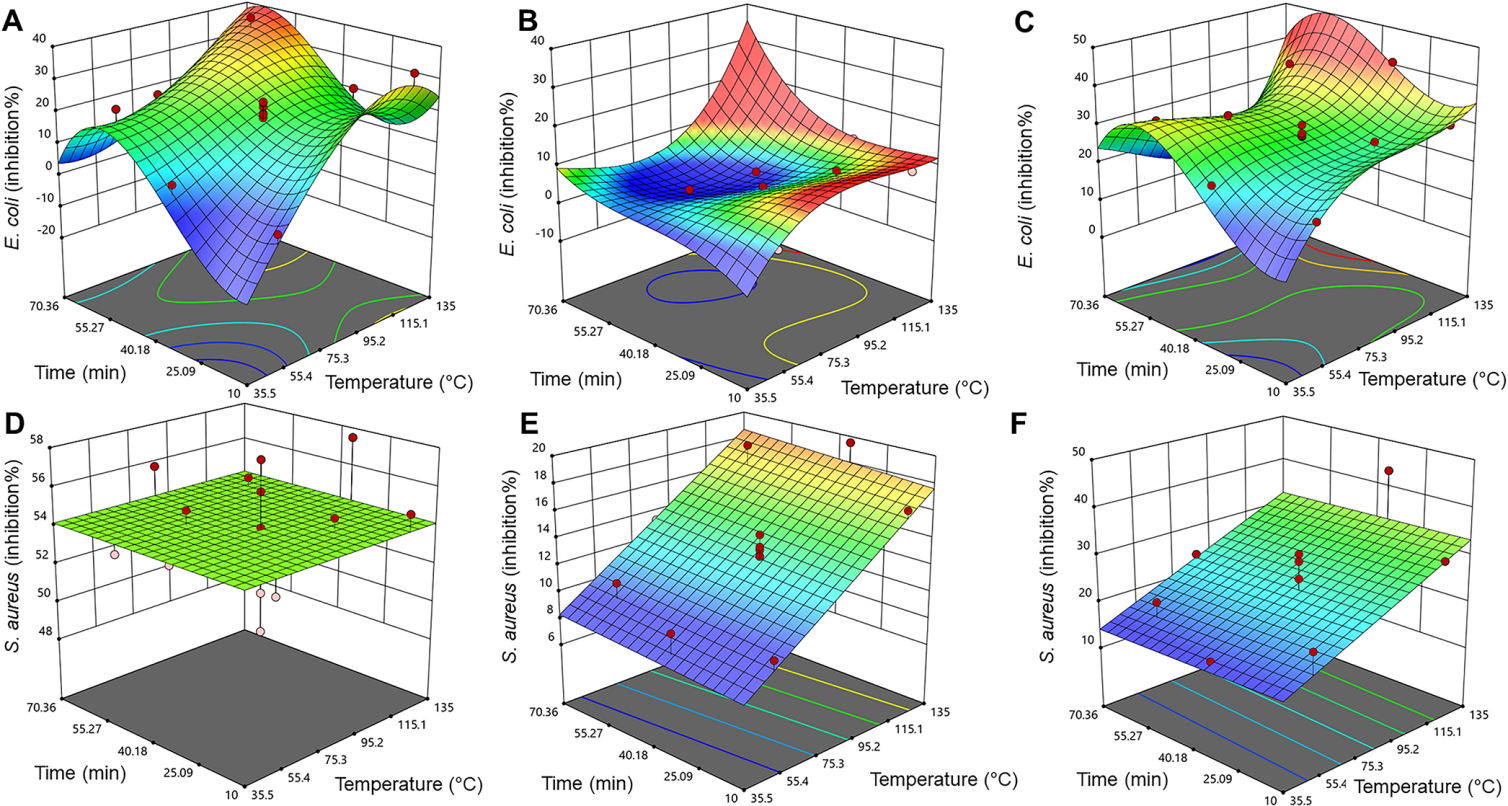
Inhibition percent value response surfaces for *E. coli* by aq. ethanol (A), water (B), and water with Na_2_CO_3_ + NaHSO_3_ addition extracts (C), and *S. aureus* inhibition percent in aq. ethanol (D), water (E), and water with Na_2_CO_3_ + NaHSO_3_ addition extracts (F). The RSM was cubic for A (*R*^2^ = 0.7753), B (*R*^2^ = 0.8438), and C (*R*^2^ = 0.7403), whereas it was mean for D (*R*^2^ = none) and linear for E (*R*^2^ = 0.8276) and F (*R*^2^ = 0.3512).

Overall, the obtained antibacterial results are typical for unpurified extracts in the case of the aq. ethanol and water with Na_2_CO_3_ + NaHSO_3_ addition extracts with *E. coli*, from 0.9% to 40% inhibition, and *S. aureus*, from 15.5% to 57.6% inhibition. A similar range of antibacterial results has been obtained, for example, in the case of unpurified bark extracts using the same bacterial method by Välimaa et al.^70^ For the water extracts, the results are low, 3.4–11.1% for *E. coli* and 9.6–19.5% for *S. aureus*, but comparable to those received using the same bacterial method for *E. coli* with pure α- and β-pinene by Muilu-Mäkelä et al.^71^ with 0.8–1.6 mg/mL concentration. For *S. aureus*, the results obtained in this study are lower, suggesting that the used 1 mg/mL concentration of the samples is insufficient for unpurified extraction products. Unpurified extraction products also likely contain carbohydrates, which could serve as a nutrient source for the bacteria. In the bacterial test results of aq. ethanol, water, and water with Na_2_CO_3_ + NaHSO_3_ addition extracts (Figure 4), it is evident that variation at the center point is small for all but *S. aureus* and aq. ethanol extracts (Figure 4D, discarded from the optimization) and *S. aureus* and water with Na_2_CO_3_ + NaHSO_3_ addition extracts (Figure 4F). The variation in these result surfaces suggests that the *S. aureus* strain is more sensitive to the effect caused by the solvents than *E. coli*. Limonene extracts could not be tested against bacteria due to the strong antibacterial activity of the solvent itself.^71^ Coniferous species have been shown to harbor various compounds with broad-acting antimicrobial properties including volatiles such as terpenoids, polyphenolic compounds, and piperidine alkaloids.^71−77^ In the response surface model for water extracts with *E. coli* (Figure 4B), there is a sink at the approximate middle point of the surface. Therefore, even though the coefficient of determination for the surface is desirable (*R*^2^ = 0.8438), this model was not considered for optimization due to its unusual behavior, likely caused by an overcompensation by the cubic model.

### Condensed Tannin Content

**Figure 5:**
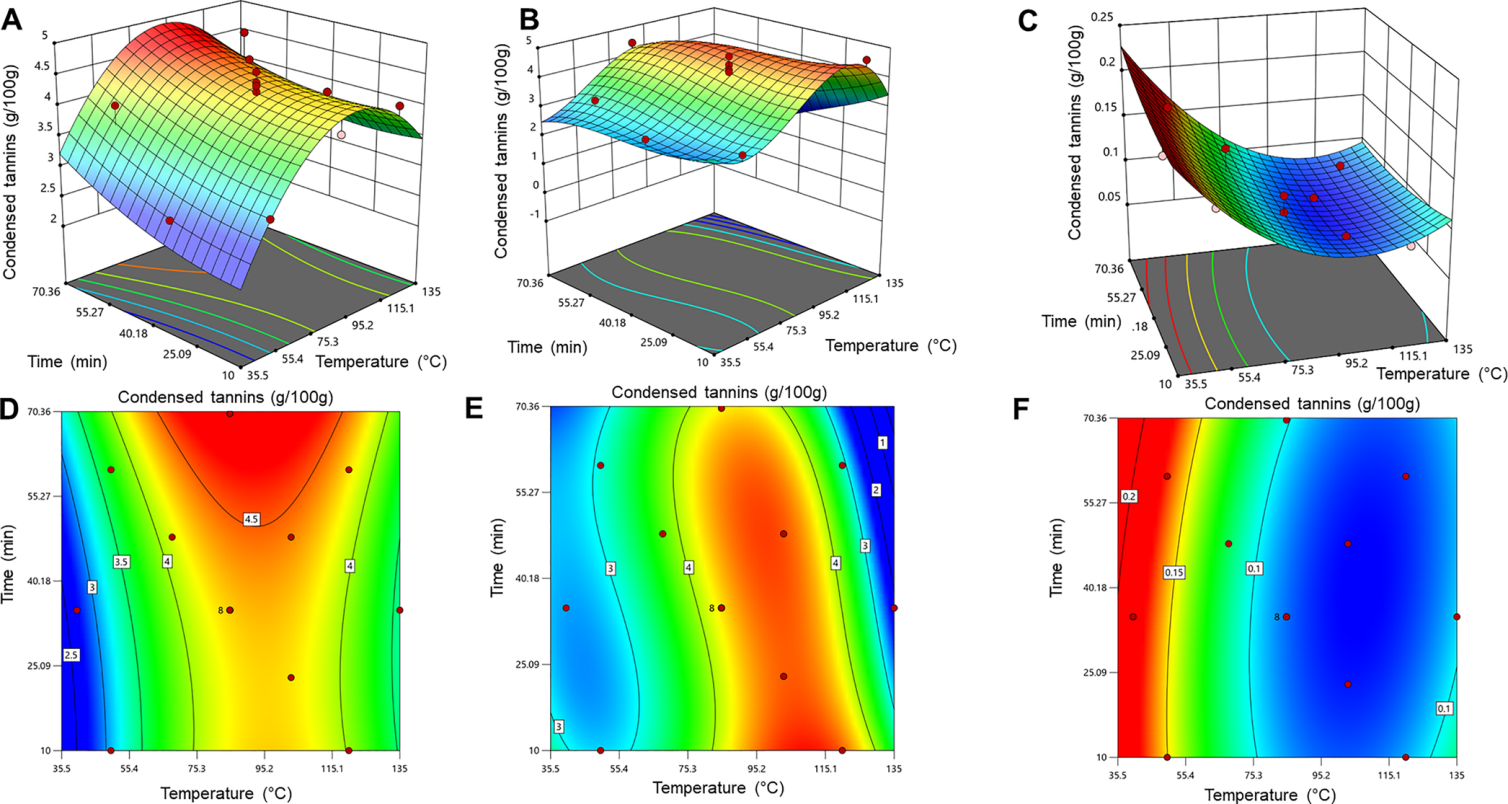
Three-dimensional surface (A, B, and C) and contour graphs (D, E, and F) depicting the yield of condensed tannins (g/100 g of dry extract) for aq. ethanol (A, D), water (B, E), and water with Na_2_CO_3_ + NaHSO_3_ addition (C, F). The RSM was quadratic for A (*R*^2^ = 0.7769), cubic for B (*R*^2^ = 0.9310), and quadratic for C (*R*^2^ = 0.8399).

Condensed tannins (Figure 5) in spruce logging residues were mixtures of procyanidins and prodelphinidins, consistent with previous studies on spruce bark and needles.^48−50,78^ The yield of condensed tannins was dependent on the extraction time and temperature. Interestingly, there were high-yield ridges at the 90–100 °C temperature range in water and aq. ethanol extraction. In that area, increasing extraction time increased yield slightly for aq. ethanol extractions. For water extractions, the highest yield area is in the temperature range of 90–110 °C, and prolonged extraction time did not improve the yield. It has been proven that rather short extraction times may be preferable when pressurized hot water is applied for the extraction of tannins and other polyphenols to avoid thermal degradation of these compounds.^79^ The extractions with sodium salts, i.e., water with Na_2_CO_3_ + NaHSO_3_, modified the tannin structure, yielding sulfonated derivatives, resulting in a decrease in the native forms of condensed tannins when time and temperature increased. Thus, in the water with Na_2_CO_3_ + NaHSO_3_ addition extracts, the highest free tannin yields were obtained with the lowest extraction temperatures. Unfortunately, it was not possible to determine the sulfonated tannin derivatives with the applied determination method.

### Optimized Conditions

**Figure 6:**
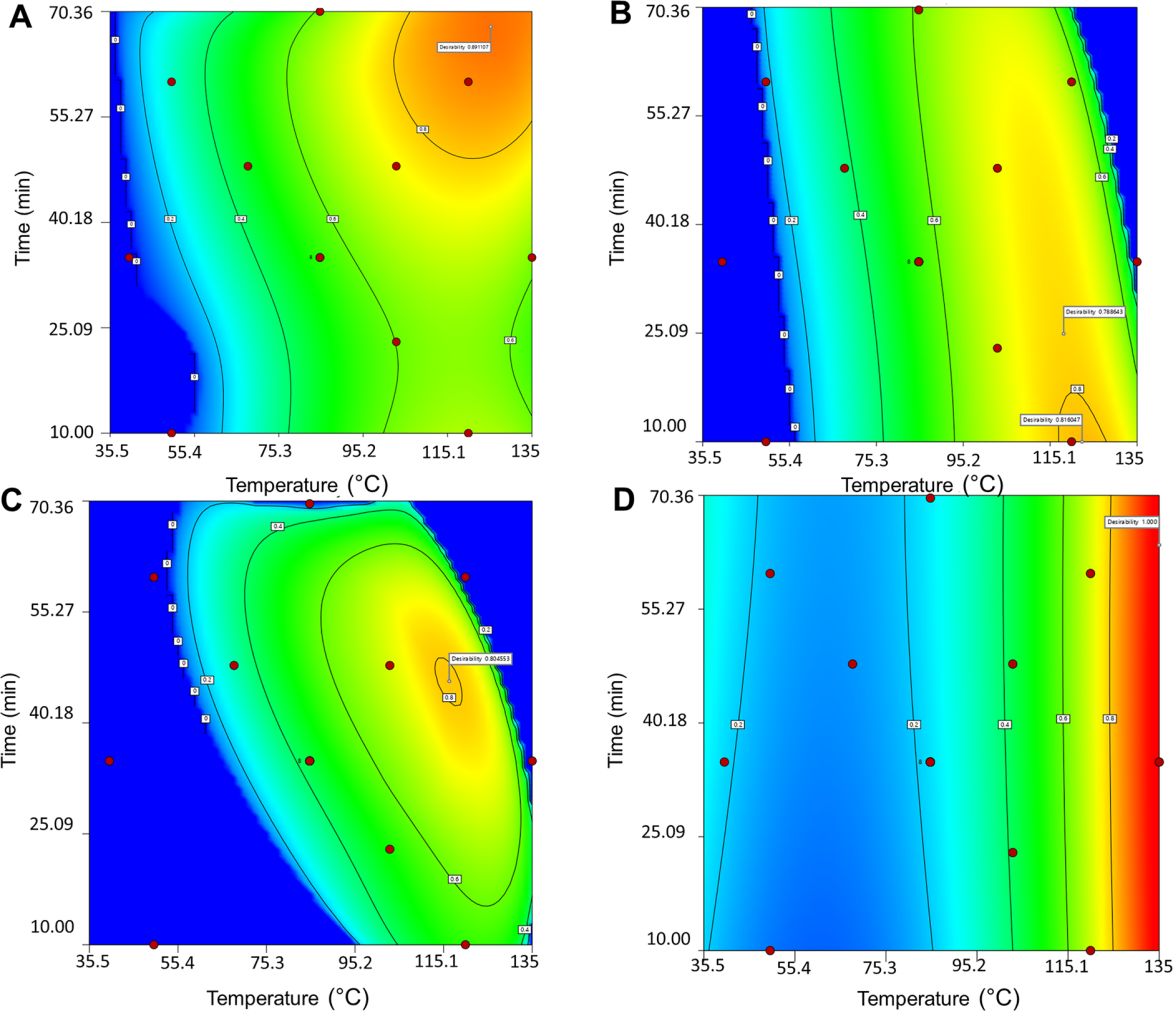
Desirability areas in contour plots for aq. ethanol (A), water (B), water with Na_2_CO_3_ + NaHSO_3_ addition (C), and limonene (D) extraction.

Since the goal was to obtain the most promising bioactive extracts, antioxidant activity, antibacterial properties, phenolic content, and condensed tannins responses were chosen to have the highest importance (3), if their adjusted coefficient of determination (*R*^2^) values were over 0.7, they were statistically significant in the 5% (*p* < 0.05) level of or lower, and their adequate precision (or signal-to-noise ratio) was over 4, with their lack of fit values being statistically insignificant (*p* > 0.1). If these criteria were not met, the response was not considered for the optimization. A high extraction yield (TDS) without bioactivity was not desired and thus not considered for optimization. Also, the composition of condensed tannins (procyanidins or prodelphinidins) was not considered crucial for the optimization. In total, four RS models were created to predict the effects of temperature and time on the extraction of logging residues for different solvents (i.e., water, water with Na_2_CO_3_ + NaHSO_3_, aq. ethanol, and limonene). Optimization results and chosen responses are shown in Table 2, and contour plots of the desirability areas are displayed in Figure 6. The relatively low temperature range from 40 to 135 °C and short extraction times from 10 to 70 min were chosen for energy preservation purposes, as less energy is used for heating, and extraction could be performed under the solvent boiling point to potentially avoid the need for a pressurized vessel. However, the results showed that the optimized temperatures exceeded the boiling point for all solvents. Generally, considering hydrolytic, oxidative, and isomerization reactions, temperatures under 100 °C are preferable. At higher temperatures (>150 °C), structural polymers such as hemicellulose and lignin start to be hydrolyzed and extracted. The aim was to obtain bioactive compounds mainly present in the cellular matrix. In addition, it has been shown that a prolonged extraction time can decrease the yield of polyphenolic and antioxidant compounds.^80^ Detailed information on the analysis of variance, sum of squares, degrees of freedom, mean squares, *F* values, and *p* values of the fitted models for all four solvent choices can be found in the Supporting Information (Supplementary Tables 1–4).

**Table 2:** Optimized Extraction Conditions for Aqueous Ethanol, Water, Water with Na_2_CO_3_ + NaHSO_3_, and Limonene

extraction solvent	temperature (°C)	time (min)	desirability	responses
aq. ethanol	125	68	0.891	5 (TPC, FRAP, ORAC, *E. coli*, CT)
water	120	10	0.826	5 (TPC, ORAC, *S. aureus*, CT, DP)
water with Na_2_CO_3_ + NaHSO_3_	111	49	0.890	3 (ORAC, *E. coli*, DP)
limonene	135	41	1.000	1 (DPPH)

Based on the optimization, aqueous ethanol extraction should be carried out at 125 °C for 68 min (Table 2) to obtain an extract with the desired properties. The optimum values for water extraction were 120 °C and 10 min, resulting in a similar temperature range as with aqueous ethanol but a reduced extraction time. The difference can be explained by the high yield of condensed tannins in the earlier phase of water extraction (Figure 5A and 5B). For water extraction with Na_2_CO_3_ + NaHSO_3_ addition, the optimal temperature was 111 °C and the extraction time was 49 min, yielding a temperature range quite similar to that of water and aq. ethanol. Interestingly, the optimized extraction time fell between the values of aq. ethanol and water extractions. For water and water with Na_2_CO_3_ + NaHSO_3_ addition, higher temperatures and extraction times were not preferred (upper right corner, Figure 6B and 6C). In contrast, with aq. ethanol extraction (Figure 6A), optimized conditions were near the edge of the highest temperature and time values, indicating differences between solvents and their applications.

Optimized conditions (135 °C and 41 min) for limonene extraction were primarily dependent on the extraction temperature (Figure 6D). Unlike other solvents, extraction time was not a critical factor in determining the optimal conditions. Only one of the responses met the requirements for consideration in the optimization of limonene extraction. There was evident heterogeneity in the industrially assorted needle-rich logging residue fraction from chipped branches (Figure 2). In addition to needles, various wood, bark, and twig parts were present, creating a complex biological matrix for extraction. Despite this complexity, the extraction optimization was successfully performed, and the theoretical optimization solutions are presented in Table 3.

**Table 3:** Theoretical Optimization Solutions Obtained with the Design Expert Software^a^

solvent	TDS, W-%	TDS, mg/g	FRAP, μM Fe(II) eq/g	ORAC, μM TE/g	TPC, mg GAE/g	*E. coli*, inh %	*S. aureus*, inh %	CT, g/100 g	DP	PC, %	PD, %
aq. EtOH	3.4	241	**412**	**2664**	**14**	**36**	54	**4.2**	3.5	96	4
water	2.1	162	303	**1933**	**11**	12	**16**	**4.6**	**3.8**	97	3
water with Na_2_CO_3_ + NaHSO_3_	2.3	186	977	**4280**	19	**34**	28	0.1	**2.2**	100	0

solvent	TDS, W-%	TDS, mg/g	CUPRAC, mg AA eq/g	DPPH, μM AA eq/g	TPC, mg GAE/g						
limonene	0.9	63	317	**2.6**	334						

a The responses used in the optimization are in bold.

In the optimized conditions (Table 3), water and water with Na_2_CO_3_ + NaHSO_3_ addition yielded a lower theoretical overall extraction yield compared to aq. ethanol. The poor TDS yield of limonene is mainly attributed to its ability to extract nonpolar compounds. The high yield for aq. ethanol could be explained by the solvent’s properties, allowing it to dissolve both polar and nonpolar compounds, such as waxes. The hydrophobic epicuticular waxes in the needles may hinder water permeability during extraction, resulting in lower TDS values. Water extraction with chemical addition showed the highest antioxidant (FRAP and ORAC) and TPC values compared to other solvents. This can be partly explained by the pH differences induced by the solvents. While water extracts yielded acidic pH values from 4.11 to 4.31, water extraction with Na_2_CO_3_ + NaHSO_3_ addition generated alkaline extracts with pH values between 8.95 and 9.85. Many of the used bioactivity tests are sensitive to pH changes, and alkaline extraction products can partly explain the higher activity results. Aqueous ethanol extracts exhibited the highest antibacterial activity in the model, while water yielded the lowest. Water would provide the highest yield for obtaining condensed tannins, with aq. ethanol showing similar values. Water extraction with Na_2_CO_3_ + NaHSO_3_ addition yielded sulfonated tannins, explaining the low value in the model.

Despite the evident heterogeneity, we were able to extract antioxidant and antibacterial products from industrially feasible starting material. The expected phenolic capacities were comparable to other wood-based extracts, such as 12 mg GAE/g (TPC) of Norway spruce bark with hot water extraction.^64^ The expected antioxidant ORAC values were also proportionate and, in some cases, even higher than those in a study by Jyske et al.,^15^ where ORAC values for freshly frozen pure needle biomass yielded approximately 1 × 10^3^ μM TE/g for hot-water extraction and 2 × 10^3^ μM TE/g for ethanol–water (70:30) extraction. However, the theoretical FRAP values in our study were approximately one-half of those obtained by Jyske et al.,^15^ possibly due to differences in the sample heterogeneity. Additionally, the optimized theoretical antibacterial activities were comparable to unpurified natural extracts of spruce bark, as demonstrated by Välimaa et al.^70^ The only exception is the expected antibacterial activity for water extraction, which is low for *S. aureus* and is attributed to the extracts containing carbohydrates that can serve as nutrition for the bacteria. While it is effortless to find suitable comparable studies for aq. ethanol and water extraction, there are limited studies available for less traditional solvents like water with chemical addition and limonene. However, Kilpeläinen et al.^40^ found that sodium carbonate addition improved the spruce bark hot water extraction yield in temperatures within 60–90 °C. The findings of this study exhibit a similar trend with Na_2_CO_3_ + NaHSO_3_ addition, where the alkalic pH can, at least partly, contribute to the increase in the antioxidant and antibacterial activities. Limonene itself has been found to possess both antioxidant and antibacterial properties and is primarily used for the extraction of nonpolar compounds such as lipids.^69,71^ For instance, pressurized limonene extraction was considered an interesting solvent alternative for extracting lipids from marine algae, with extraction efficiency dependent on the microalgae species chosen.^81^ However, in this study, our focus was on bioactivity maximization. While investigating the lipid profiles would have been interesting, it was beyond the scope of this study. Bioactive lipids, although existing, typically do not exhibit their highest potential in terms of antioxidant and antibacterial properties. Therefore, unsurprisingly, limonene extracts yielded the lowest expected bioactivities in this study.

Verification of the theoretical results was performed for aqueous ethanol under the extraction conditions of 110 °C and 60 min, and the theoretical and experimental results are presented in the Supporting Information (Supplementary Table 6). The verification reveals that within the 95% tolerance interval for a 99% population, TDS, yield, ORAC, TPC, and *E. coli* results fall between the highest and the lowest predicted values. However, values for FRAP and *S. aureus* did not fit between the tolerance intervals. This further supports our hypothesis that the FRAP test is more sensitive to potential changes in sampling, assortment, and handling procedures, leading to variation in the heterogeneous biomass constitution. The RSM for *S. aureus* (Figure 4D) demonstrates that the strain is too sensitive to the solvent itself to provide proper values in the optimization, and it was not considered for constructing the desirability surfaces. Therefore, it is evident that the optimization could be verified as successful as can be expected for industrially assorted and heterogeneous sample biomass.

## Conclusions

In this study, we present a biorefinery-inspired option for the higher potential utilization of needle-rich logging residues. The study demonstrates that the extraction of industrially assorted spruce logging residues to obtain antioxidant and antibacterial fractions is feasible and can be successfully optimized. The optimized extraction conditions were 125 °C and 68 min for aqueous ethanol, 120 °C and 10 min for water, 111 °C and 49 min for water with Na_2_CO_3_ + NaHSO_3_ addition, and 134 °C and 41 min for limonene using 5, 5, 3, and 1 of the responses, respectively. Unlike many existing studies, multiple target variables were simultaneously considered using RSM, providing the opportunity to obtain valuable extracts with a high concentration of condensed tannins and total phenolics exhibiting antioxidant and antibacterial properties. Under the optimized conditions, aqueous ethanol extraction resulted in a higher overall yield (241 mg/g) with increased antioxidant activities (FRAP 412 μM Fe(II) eq/g and ORAC 2664 μM TE/g) and bacterial inhibition (36% against *E. coli* and 54% against *S. aureus*) compared to water (yield 303 mg/g; FRAP 303 μM Fe(II) eq/g; ORAC 1933 μM TE/g; 12% inhibition against *E. coli* and 16% against *S. aureus*). Chemical addition to water produced extracts with even higher antioxidant values than those with aqueous ethanol (FRAP 977 μM Fe(II) eq/g; ORAC 4280 μM TE/g) but likely resulted in sulfonated condensed tannins, leading to a decrease in CT from 4.2 g/100 g in aqueous ethanol to 0.1 g/100 g in water with Na_2_CO_3_ + NaHSO_3_ extraction. In this work, while industrial scale was used for the logging residue assortment, laboratory-scale extraction was employed for the optimization process. The optimized extraction conditions can also be scaled up for industrial use, which, however, remains a prospect for future work.

## Supporting Information Available

Detailed method descriptions for individual responses; mathematical modeling; optimized factors; two-factor central composite quadratic design used for optimization; analysis of variance for the fitted models; individual responses used for the optimization; theoretical and experimental results at conditions 110 °C and 60 min; RSM responses for total dissolved solids; RSM responses for total phenolic content; RSM responses for antioxidant activity for water with Na_2_CO_3_ + NaHSO_3_ and limonene extracts (PDF)

## Abbreviations

AA eq: ascorbic acid equivalent
ASE: accelerated solvent extraction
aq.: aqueous
CT: condensed tannins
CUPRAC: cupric ion reducing antioxidant capacity
DOE: design of experiment
DP: degree of polymerization
DPPH: 2,2-diphenyl-1-picrylhydrazyl radical
DW: dry weight
eq: equivalent
2FI: two-factor interaction
FRAP: ferric reducing antioxidant potential
GAE: gallic acid equivalent
HAT: hydrogen-atom transfer
LA: lysogeny agar
ORAC: oxygen radical absorbance capacity
PB: phosphate buffer
PC: procyanidin
PD: prodelphinidin
RLU: relative light unit
RSM: response surface methodology
SET: single electron transfer
TDS: total dissolved solids
TE: Trolox equivalent
TPC: total phenolic content
TPTZ: 2,4,6-tris(2-pyridyl)-*s*-triazine
