# Supplementary material for: Valorizing Assorted Logging Residues: Response Surface Methodology in the Extraction Optimization of a Green Norway Spruce Needle-Rich Fraction To Obtain Valuable Bioactive Compounds
Source: ACS Sustain Resour Manag. 2024 Feb 2;1(2):237–49. doi: 10.1021/acssusresmgt.3c00050 (PMC10895920; doi:10.1021/acssusresmgt.3c00050)
Supplement: Supplementary file 1 — rm3c00050_si_001.pdf [file rm3c00050_si_001.pdf]

Supporting Information:

Valorizing assorted logging residues: Response surface methodology in the extraction optimization of green Norway spruce needle-rich fraction to obtain valuable bioactive compounds

AUTHOR NAMES

Jenni Tienaho, *[†] *Marina Fidelis,* [†‡] *Hanna Brännström,* [II] *Jarkko Hellström,* [III] *Magnus Rudolfsson,* [IV] *Atanu Kumar Das,* [IV] *Jaana Liimatainen,* [†] *Anuj Kumar,* [†] *Mika Kurkilahti,* [§] *Petri Kilpeläinen* [†]

AUTHOR ADDRESS

[†] Production Systems, Natural Resources Institute Finland (Luke), Latokartanonkaari 9, FI-00790 Helsinki, Finland

[‡] Food Sciences Unit, Department of Life Technologies, University of Turku, FI-20014 Turku, Finland

[II] Production Systems, Natural Resources Institute Finland (Luke), Teknologiakatu 7, FI-67100 Kokkola, Finland

[III] Production Systems, Natural Resources Institute Finland (Luke), Myllytie 1, FI-31600 Jokioinen, Finland

[IV] Swedish University of Agricultural Sciences, Unit of Biomass Technology and Chemistry, SE-901 83 Umeå, Sweden

[§] Natural Resources, Natural Resources Institute Finland (Luke), Itäinen Pitkäkatu 4 A, FI-20520 Turku, Finland

Contents:

Supplementary Document 1: Detailed method descriptions for individual responses; Mathematical modeling; Optimized factors; Two-factor central composite quadratic design used for optimization.

Supplementary Table 1-4: Analysis of variance for the fitted models with water (1); aqueous ethanol (2); water with Na_2CO_3+$NaHSO_3$ addition (3) and limonene (4) extraction.

Supplementary Table 5: Individual responses used for the optimization.

Supplementary Table 6: The theoretical and experimental results at conditions 110 °C and 60 min.

Supplementary Figure 1: RSM responses for total dissolved solids (TDS) in Weight-%.

Supplementary Figure 2: RSM responses for total phenolic content (TPC) in mg GAE/g.

Supplementary Figure 3: RSM responses for antioxidant activity for water with Na_2CO_3+$NaHSO_3$ and limonene extracts.

Supplementary Document 1: Detailed method descriptions for individual responses; Mathematical modeling; Optimized factors; Two-factor central composite quadratic design used for optimization.

TDS

TDS was determined in 3 mL aliquots of extract duplicates after overnight oven-drying at 105 °C. To avoid the formation of a hardened surface layer preventing evaporation during drying, a small pre-weighed amount of sand was used to break the surface tension with limonene extracts.

Antioxidant analyses

The Ferric Reducing Antioxidant Power (FRAP) method is based on antioxidant's capacity to reduce chelated metal ions (Fe^{3+}) via a single electron transfer (SET) mechanism. In the presence of antioxidants, $[Fe(III)(TPTZ)_2]^{3+}$ is reduced and turns to reveal a deep blue color. The methodology is slightly modified from the one reported by Benzie & Strain [1]. Three technical replicates of 25 µL were investigated in a microplate format as reported by Tienaho et al. [2]. In addition to the sample, the reaction mixture contained 20 mM $FeCl_3 \bullet 6H_2O$ and 10 mM 2,4,6-Tris(2-pyridyl)-s-triazine (TPTZ) in 300 mM acetate buffer (pH 3.6). The absorbance of the reaction mixture was measured at 593 nm with a microplate reader (Varioskan Flash, Thermo Scientific) to monitor the formation of ferrous-tripyridyltriazine complex. A dilution series of $FeSO_4 \bullet 7H_2O$ was used as a standard compound against which all the results were compared. L(+)-ascorbic acid (150 µM and 800 µM) (VWR Chemicals) was used as a positive control, and the results are expressed as µM Fe(II) equivalents per gram of extracted biomass (DW).

Oxygen Radical Absorbance Capacity ($ORAC_{FL}$) is a method based on antioxidant's ability to prevent peroxyl radical from harming a fluorescent molecule, fluorescein. The method

follows the hydrogen atom transfer (HAT) mechanism and is based on the method reported by Huang et al. [3] and Prior et al. [4]. The experimental setup is explained in more detail by Tienaho et al. [5]. In brief, all the samples were measured in five dilutions, each with two technical replicates. The reaction mixture contained the sample in 0.075 M phosphate buffer (PB) at pH 7.5 (Merck) and 8.16×10^{-5} mM fluorescein as well as the peroxyl radical creator 2,2'-azobis(2-methylpropionamidine) dihydrochloride. Fluorescence (emission 538 nm; excitation 485 nm with bandwidth 12 nm) was measured with a microplate reader (Varioskan Flash, Thermo Scientific) 21 times every 2 minutes, and the microplate was briefly shaken prior to every measurement and kept at 37 °C. The vitamin E analogue, Trolox ((±)-6-Hydroxy-2,5,7,8-tetramethylchromane-2-carboxylic acid) was used as the standard substance against which all the results were compared and finally expressed as Trolox equivalents (TE) per gram of extracted biomass (µM TE / g DW).

The 2,2-Diphenyl-1-picrylhydrazyl (DPPH) free-radical scavenging assay is based on the capture of DPPH by antioxidants. Thus, its absorbance gradually decreases with time (yellow). This test protocol was performed following the method described by Brand-Williams et al. [6]. Briefly, an aliquot of 40 µL of a diluted sample and 260 µL of a 0.10 mM of DPPH methanolic solution were pipetted to a 96-well plate. The obtained mixture was left in the dark at 25 °C for 30 min. Water was used as a blank sample. After the reaction time, the decrease in DPPH absorbance was measured at 517 nm. The procedure was performed in triplicate, and the results were expressed as mg of ascorbic acid equivalent (AAE) per gram of extracted biomass (mg AAE/ g DW).

The Cupric Ion-Reducing Antioxidant Capacity (CUPRAC) was used to evaluate the antioxidant activity of the extracts based on an electron transfer mechanism by utilizing the copper (II) neocuproine (Cu(II)-Nc) reagent as the chromogenic oxidant [7]. In short, 100 µL of the ascorbic acid solution, diluted sample, or blank (water), were mixed with 1 mL of each

solution into a test tube: $CuCl_2 \bullet 2H_2O$ (1.0×10^{-2} M), neocuproin (7.5×10^{-3} M) solution, NH_4Ac (1 M, pH 7.0 buffer), and ultrapure water to make up the final volume (i.e., 4.1 mL). Absorbance was recorded at 450 nm against a blank (ultrapure water) after 30 min incubation. The test was carried out in test tubes, and final reaction mixture absorbance measured using a microplate reader (Hidex SENSE) by pipetting 300 µL of the final reaction mixture into each well and measuring it at 450 nm. The experiments were performed in triplicate, and the results expressed as mg of ascorbic acid equivalent per gram of extracted biomass (mg AAE/ g DW).

Total phenolic content (TPC)

The Prussian blue assay for TPC is based on the phenolic compound's ability to reduce chelated hexacyanoferrate (III) ion into hexacyanoferrate (II) ion, which reacts with Fe^{3+} to form a Prussian blue color. The method used has been previously reported by Margraf et al. [8]. In brief, samples were pipetted in technical triplicates and at least three dilutions into a microplate with 0.50 mM $FeCl_3 \bullet 6H_2O$ diluted in 0.01 M HCl. An aliquot of 0.50 mmol/L $K_3[Fe(CN)_6]$ solution was then injected into the microplate, and the plate was shortly shaken. Absorbance was measured at 725 nm after 15 min using a microplate reader (Varioskan Flash, Thermo Scientific). The results are expressed as mg gallic acid equivalents (GAE) per gram of extracted biomass (mg GAE / g DW).

The total reducing power of the lipophilic compounds in the limonene samples was evaluated in an alkaline medium using the **modified Folin–Ciocalteu method** by Berker et. [9]. The original method was adjusted to enable the simultaneous measurement of lipophilic and hydrophilic phenolic compounds in a NaOH-added isobutanol−water medium (Merck, Germany). In this regard, the Folin–Ciocalteu reagent (VWR Chemicals, Germany) was diluted in isobutanol at a ratio of 1:2 (v/v), and an aliquot of 75 µL was added to an aliquot of sample (50 µL) diluted in acetone (VWR Chemicals, Germany), followed by the addition of 875 µL of a 0.1 M NaOH solution and 1.5 mL of ultrapure water. The glass tubes were vortexed for 15 s

and left to react for 20 min. The absorbance of the colored complex was read at 665 nm using a microplate reader (Hidex SENSE), and the values were expressed as mg of gallic acid equivalents per gram of extracted biomass (mg GAE/g DW).

Antibacterial properties

Recombinant *Escherichia coli* K12 + pcGLS11 (gram-negative) and *Staphylococcus aureus* RN4220+pAT19 (gram-positive) bacteria were used in this study, and have been modified to produce continuous luminescent light as part of their normal metabolism (both strains were first reported by Vesterlund et al. [10]). When exposed to antibacterial substances, luminescent light production is decreased, and the reduction of the signal is dose-dependent on the antibacterial agent concentration. The storage, cultivation, and test protocol have been previously described. [2] In brief, the strains were stored at -80 °C, and stocks were initiated by approximately 16 h cultivation at 30 °C (for *E. coli*) and 37 °C (for *S. aureus*) on lysogeny agar plates (tryptone 10 g/L; yeast extract 5 g/L; NaCl 10 g/L; and agar 15 g/L). The LA plates were supplemented with 10% (v/v) sterile filtered phosphate buffer (PB) (1 M, pH 7.0) and 100 µg/mL of ampicillin for *E. coli* and with 5 µg/mL erythromycin for *S. aureus*. Biosensor stocks were prepared by inoculating a single colony of bacteria in lysogeny broth with the same supplements as plates. Stocks were cultivated for approximately 16 h at 300 rpm shaking at 30 °C (*E. coli*) and 37 °C (*S. aureus*). All samples were diluted in water to obtain three suitable concentrations to observe dose responses with the bacteria. The samples and positive (ethanol) and negative (double-distilled water) controls were pipetted in triplicates into opaque white polystyrene microplates with the same volume of bacterial inoculation. The produced luminescent light signal was then measured using a Varioskan Flash Multilabel device (Thermo Fischer Scientific) once every 5 min for 95 min at room temperature and the plate was briefly shaken before every measurement. Results are expressed as inhibition percentages (inhibition%) of 1 mg/mL of the sample after 50 min of incubation. Inhibition percentages are

calculated as follows, inhibition% = (1-RLU_{sample}/$RLU_{neg\_control}$) × 100%, where RLU is relative light units obtained from the microplate reader.

Condensed tannins determination

Condensed tannins (CT, proanthocyanins) were determined by a thiolytic degradation method according to Korkalo et al. [11]. Briefly, 20–30 mg of the sample was mixed with 1 mL of methanolic cysteamine acidified by hydrochloric acid. After 60 min incubation at 65 °C, the thiolysis reaction was terminated, and the sample solutions were filtered into HPLC vials and analyzed by UHPLC-DAD-FLD. Catechin, epicatechin, gallocatechin, epigallocatechin, and thiolyzed procyanidin B2 (Extrasynthese, Lyon, France) were used as external standards for quantification. Results are expressed as g/100g of dry extract.

Mathematical modeling

An example of the simplest model was used with the *S. aureus* test for water extraction and is informally written as (**Eq. 1**):

$$\text{Response} = A + B + \text{Block} \qquad \textbf{Eq. 1}$$

and an example of the most complicated model was used with the *E. coli* test for water extracts (**Eq.2**):

$$\text{Response} = A + B + AB + A^2 + B^2 + A^2B + AB^2 + A^3 + B^3 + \text{Block} \qquad \textbf{Eq. 2}$$

Here, A represents the temperature factor, and B represents the time factor. Detailed information on these and all the other used models can be found separately for each solvent in **Supplementary tables 1– 4**.

The temperatures ranged from 40 to 135 °C, and the extraction times from 10 to 70 min (**Table 1** and **Table 2**). The ASE system uses a constant 103.4 bar (1500 Psi) pressure for the extractions.

Table 1. Optimized factors and their coded and actual values used in creating the optimization design.

Optimized factor	Units	Actual minimum	Actual maximum	Coded Low	Coded High	Mean	Std. Dev.
Temperature	°C	40.00	135.00	35.50	120.00	86.33	24.65
Time	min	10.00	70.00	10.00	70.36	37.72	15.48

Table 2. Two-factor central composite quadratic design used for optimization in RSM. Run= Extraction order; Std = Standard order to form same run-setting designs. Two blocks were used to remove possible day-to-day variation.

Run	Std [a]	Block	Temperature (°C)	Time (min)	Central point
1	3	1	120	60	
2	6	1	85	35	*
3	18	1	40	35	
4	1	1	50	60	
5	4	1	85	35	*
6	5	1	50	10	
7	2	1	85	70	
8	13	1	135	35	
9	14	1	85	35	*
10	15	1	120	10	
11	16	1	85	35	*
12	17	1	85	35	*
13	9	2	103	48	
14	10	2	85	35	*
15	8	2	85	35	*
16	7	2	68	48	
17	12	2	103	23	
18	11	2	85	35	*

[a] *randomized*

References:

(1) Benzie, I. F. F.; Strain, J. J. The Ferric Reducing Ability of Plasma (FRAP) as a Measure of "Antioxidant Power": The FRAP Assay. *Analytical Biochemistry* **1996**, *239* (1), 70–76. https://doi.org/10.1006/abio.1996.0292.

(2) Tienaho, J.; Reshamwala, D.; Sarjala, T.; Kilpeläinen, P.; Liimatainen, J.; Dou, J.; Viherä-Aarnio, A.; Linnakoski, R.; Marjomäki, V.; Jyske, T. Salix Spp. Bark Hot Water Extracts Show Antiviral, Antibacterial, and Antioxidant Activities—The Bioactive Properties of 16

Clones. *Front. Bioeng. Biotechnol.* **2021**, *9*, 797939. https://doi.org/10.3389/fbioe.2021.797939.

(3) Huang, D.; Ou, B.; Hampsch-Woodill, M.; Flanagan, J. A.; Prior, R. L. High-Throughput Assay of Oxygen Radical Absorbance Capacity (ORAC) Using a Multichannel Liquid Handling System Coupled with a Microplate Fluorescence Reader in 96-Well Format. *J. Agric. Food Chem.* **2002**, *50* (16), 4437–4444. https://doi.org/10.1021/jf0201529.

(4) Prior, R. L.; Hoang, H.; Gu, L.; Wu, X.; Bacchiocca, M.; Howard, L.; Hampsch-Woodill, M.; Huang, D.; Ou, B.; Jacob, R. Assays for Hydrophilic and Lipophilic Antioxidant Capacity (Oxygen Radical Absorbance Capacity ($ORAC_{FL}$)) of Plasma and Other Biological and Food Samples. *J. Agric. Food Chem.* **2003**, *51* (11), 3273–3279. https://doi.org/10.1021/jf0262256.

(5) Tienaho, J.; Karonen, M.; Muilu-Mäkelä, R.; Kaseva, J.; de Pedro, N.; Vicente, F.; Genilloud, O.; Aapola, U.; Uusitalo, H.; Vuolteenaho, K.; Franzén, R.; Wähälä, K.; Karp, M.; Santala, V.; Sarjala, T. Bioactive Properties of the Aqueous Extracts of Endophytic Fungi Associated with Scots Pine (Pinus Sylvestris) Roots. *Planta Med* **2020**, *86* (13/14), 1009–1024. https://doi.org/10.1055/a-1185-4437.

(6) Brand-Williams, W.; Cuvelier, M. E.; Berset, C. Use of a Free Radical Method to Evaluate Antioxidant Activity. *LWT - Food Science and Technology* **1995**, *28* (1), 25–30. https://doi.org/10.1016/S0023-6438(95)80008-5.

(7) Apak, R.; Güçlü, K.; Özyürek, M.; Çelik, S. E. Mechanism of Antioxidant Capacity Assays and the CUPRAC (Cupric Ion Reducing Antioxidant Capacity) Assay. *Microchim Acta* **2008**, *160* (4), 413–419. https://doi.org/10.1007/s00604-007-0777-0.

(8) Margraf, T.; Karnopp, A. R.; Rosso, N. D.; Granato, D. Comparison between Folin-Ciocalteu and Prussian Blue Assays to Estimate The Total Phenolic Content of Juices and Teas Using 96-Well Microplates: Prussian Blue Assay for Total Phenols…. *Journal of Food Science* **2015**, *80* (11), C2397–C2403. https://doi.org/10.1111/1750-3841.13077.

(9) Berker, K. I.; Ozdemir Olgun, F. A.; Ozyurt, D.; Demirata, B.; Apak, R. Modified Folin–Ciocalteu Antioxidant Capacity Assay for Measuring Lipophilic Antioxidants. *J. Agric. Food Chem.* **2013**, *61* (20), 4783–4791. https://doi.org/10.1021/jf400249k.

(10) Vesterlund, S.; Paltta, J.; Lauková, A.; Karp, M.; Ouwehand, A. Rapid Screening Method for the Detection of Antimicrobial Substances. *Journal of Microbiological Methods* **2004**, *2004* (57), 23–31. https://doi.org/10.1016/j.mimet.2003.11.014.

(11) Korkalo, P.; Korpinen, R.; Beuker, E.; Sarjala, T.; Hellström, J.; Kaseva, J.; Lassi, U.; Jyske, T. Clonal Variation in the Bark Chemical Properties of Hybrid Aspen: Potential for Added Value Chemicals. *Molecules* **2020**, *25* (19), 4403. https://doi.org/10.3390/molecules25194403.

Supplementary Table 1. Analysis of variance for the fitted models - water

Water	Source	Sum of Squares (SS)	Degree of freedom (df)	Mean Square (MS)	*F*-value	*p*-value
**TDS**	Block	0.0029	1	0.0029		
	Model	1.61	5	0.3222	139.45	< 0.0001
	A-Temperature	0.7258	1	0.7258	314.15	< 0.0001
	B-Time	0.1805	1	0.1805	78.12	< 0.0001
	AB	0.0008	1	0.0008	0.3351	0.5743
	A^2	0.0589	1	0.0589	25.49	0.0004
	B^2	0.0433	1	0.0433	18.72	0.0012
	Residual	0.0254	11	0.0023		
	Lack of Fit	0.0096	5	0.0019	0.7286	0.6273
	Pure Error	0.0158	6	0.0026		
	Cor Total	1.64	17			
	Std. Dev.	0.0481				
	Mean	1.95				
	C.V. %	2.47				
	R^2	0.9845				
	Adjusted R^2	0.9774				
	Predicted R^2	0.9577				
	Adeq Precision	46.6111				
**FRAP**	Block	524.41	1	524.41		
	Model	46275.42	2	23137.71	97.45	< 0.0001
	A-Temperature	43704.27	1	43704.27	184.08	< 0.0001
	B-Time	3495.13	1	3495.13	14.72	0.0018
	Residual	3323.89	14	237.42		
	Lack of Fit	2733.18	8	341.65	3.47	0.0734
	Pure Error	590.71	6	98.45		
	Cor Total	50123.71	17			
	Std. Dev.	15.41				
	Mean	257.6				
	C.V. %	5.98				
	R^2	0.933				
	Adjusted R^2	0.9234				
	Predicted R^2	0.8798				
	Adeq Precision	27.3164				
S. aureus	Block	2.44	1	2.44		
	Model	111.26	2	55.63	39.41	< 0.0001
	A-Temperature	110.88	1	110.88	78.55	< 0.0001
	B-Time	1.08	1	1.08	0.767	0.3959
	Residual	19.76	14	1.41		
	Lack of Fit	7.78	8	0.9724	0.4869	0.8295
	Pure Error	11.98	6	2		
	Cor Total	133.47	17			
	Std. Dev.	1.19				
	Mean	13.23				
	C.V. %	8.98				
	R^2	0.8492				
	Adjusted R^2	0.8276				
	Predicted R^2	0.7847				
	Adeq Precision	17.6136				
E. coli	Block	0.6697	1	0.6697		
	Model	124.09	9	13.79	10.61	0.0026

	A-Temperature	1.49	1	1.49	1.15	0.3193
	B-Time	19.35	1	19.35	14.88	0.0062
	AB	1.19	1	1.19	0.9157	0.3705
	A^2	0.5171	1	0.5171	0.3978	0.5483
	B^2	17.58	1	17.58	13.53	0.0079
	A^2B	17.93	1	17.93	13.79	0.0075
	AB^2	8.39	1	8.39	6.45	0.0386
	A^3	4.77	1	4.77	3.67	0.097
	B^3	7.35	1	7.35	5.65	0.0491
	Residual	9.1	7	1.3		
	Lack of Fit	4.42	1	4.42	5.67	0.0546
	Pure Error	4.68	6	0.7794		
	Cor Total	133.86	17			
	Std. Dev.	1.14				
	Mean	5.99				
	C.V. %	19.03				
	R^2	0.9317				
	Adjusted R^2	0.8438				
	Predicted R^2	NA[(1)]				
	Adeq Precision	8.9513				
**ORAC**	Block	47394	1	47394		
	Model	1.69E+06	2	8.46E+05	21.11	< 0.0001
	A-Temperature	1.69E+06	1	1.69E+06	42.21	< 0.0001
	B-Time	3860.08	1	3860.08	0.0963	0.7609
	Residual	5.61E+05	14	40076		
	Lack of Fit	4.27E+05	8	53371.54	2.39	0.1524
	Pure Error	1.34E+05	6	22348.6		
	Cor Total	2.30E+06	17			
	Std. Dev.	200.19				
	Mean	1512.47				
	C.V. %	13.24				
	R^2	0.751				
	Adjusted R^2	0.7154				
	Predicted R^2	0.5281				
	Adeq Precision	12.912				
**TPC (PB)**	Block	1.41	1	1.41		
	Model	84.68	2	42.34	22.22	< 0.0001
	A-Temperature	80.1	1	80.1	42.03	< 0.0001
	B-Time	6.25	1	6.25	3.28	0.0916
	Residual	26.68	14	1.91		
	Lack of Fit	15.01	8	1.88	0.9644	0.533
	Pure Error	11.67	6	1.95		
	Cor Total	112.77	17			
	Std. Dev.	1.38				
	Mean	8.98				
	C.V. %	15.38				
	R^2	0.7604				
	Adjusted R^2	0.7262				
	Predicted R^2	0.6115				
	Adeq Precision	13.0347				
**CT**	Block	0.2179	1	0.2179		
	Model	8.16	9	0.9064	24.98	0.0002
	A-Temperature	0.9676	1	0.9676	26.67	0.0013

	B-Time	0.0877	1	0.0877	2.42	0.164
	AB	0.1214	1	0.1214	3.35	0.1101
	A^2	0.3806	1	0.3806	10.49	0.0143
	B^2	0.0238	1	0.0238	0.6547	0.4451
	A^2B	0.1618	1	0.1618	4.46	0.0726
	AB^2	0.0139	1	0.0139	0.3826	0.5558
	A^3	0.6098	1	0.6098	16.81	0.0046
	B^3	0.0615	1	0.0615	1.7	0.2341
	Residual	0.254	7	0.0363		
	Lack of Fit	0.023	1	0.023	0.5967	0.4692
	Pure Error	0.231	6	0.0385		
	Cor Total	8.63	17			
	Std. Dev.	0.1905				
	Mean	3.97				
	C.V. %	4.8				
	R^2	0.9698				
	Adjusted R^2	0.931				
	Predicted R^2	NA[(1)]				
	Adeq Precision	16.6292				
**CT (DP)**	Block	0.1122	1	0.1122		
	Model	0.5875	9	0.0653	14.54	0.001
	A-Temperature	0.0968	1	0.0968	21.56	0.0024
	B-Time	0	1	0	0.007	0.9356
	AB	0.0209	1	0.0209	4.65	0.0679
	A^2	0.0011	1	0.0011	0.2368	0.6414
	B^2	0.0087	1	0.0087	1.94	0.2064
	A^2B	0.004	1	0.004	0.8839	0.3784
	AB^2	0.0055	1	0.0055	1.22	0.3056
	A^3	0.105	1	0.105	23.39	0.0019
	B^3	0.0003	1	0.0003	0.0766	0.7899
	Residual	0.0314	7	0.0045		
	Lack of Fit	0.0003	1	0.0003	0.056	0.8209
	Pure Error	0.0311	6	0.0052		
	Cor Total	0.7312	17			
	Std. Dev.	0.067				
	Mean	3.54				
	C.V. %	1.89				
	R^2	0.9492				
	Adjusted R^2	0.8839				
	Predicted R^2	NA[(1)]				
	Adeq Precision	16.4285				

[(1)] Case(s) with leverage of 1.0000: Pred R^2 and PRESS statistic not defined.

Supplementary Table 2. Analysis of variance for the fitted models - aqueous ethanol

Ethanol	Source	Sum of Squares (SS)	Degree of freedom (df)	Mean Square (MS)	*F* -value	*p* -value
**TDS**	Block	0.0301	1	0.0301		
	Model	3.43	5	0.6857	128.02	< 0.0001
	A-Temperature	2.63	1	2.63	490.77	< 0.0001
	B-Time	0.4469	1	0.4469	83.43	< 0.0001
	AB	0.003	1	0.003	0.5537	0.4724
	A²	0.4541	1	0.4541	84.78	< 0.0001
	B²	0.0209	1	0.0209	3.91	0.0736
	Residual	0.0589	11	0.0054		
	Lack of Fit	0.0264	5	0.0053	0.9726	0.5018
	Pure Error	0.0325	6	0.0054		
	Cor Total	3.52	17			
	Std. Dev.	0.0732				
	Mean	2.81				
	C.V. %	2.6				
	R^2	0.9831				
	Adjusted R^2	0.9754				
	Predicted R^2	0.9328				
	Adeq Precision	37.6585				
**FRAP**	Block	1211.04	1	1211.04		
	Model	69667.98	5	13933.6	100.16	< 0.0001
	A-Temperature	58243.35	1	58243.35	418.68	< 0.0001
	B-Time	4900.31	1	4900.31	35.23	< 0.0001
	AB	40.81	1	40.81	0.2934	0.5989
	A²	7786.42	1	7786.42	55.97	< 0.0001
	B²	387.65	1	387.65	2.79	0.1232
	Residual	1530.24	11	139.11		
	Lack of Fit	828.16	5	165.63	1.42	0.339
	Pure Error	702.09	6	117.01		
	Cor Total	72409.26	17			
	Std. Dev.	11.79				
	Mean	319.6				
	C.V. %	3.69				
	R^2	0.9785				
	Adjusted R^2	0.9687				
	Predicted R^2	0.9578				
	Adeq Precision	33.0123				
**ORAC**	Block	250400	1	250400		
	Model	2835000	2	1418000	24.68	< 0.0001
	A-Temperature	2785000	1	2785000	48.49	< 0.0001
	B-Time	84878.83	1	84878.83	1.48	0.2442
	Residual	804100	14	57435.34		
	Lack of Fit	611000	8	76377.43	2.37	0.1541
	Pure Error	193100	6	32179.22		
	Cor Total	3890000	17			
	Std. Dev.	239.66				
	Mean	1934.22				
	C.V. %	12.39				
	R^2	0.7791				
	Adjusted R^2	0.7475				
	Predicted R^2	0.5341				
	Adeq Precision	13.8394				
**TPC (PB)**	Block	0.0872	1	0.0872		
	Model	72.7	2	36.35	42.12	< 0.0001
	A-Temperature	63.66	1	63.66	73.77	< 0.0001
	B-Time	11.07	1	11.07	12.82	0.003

	Residual	12.08	14	0.8629		
	Lack of Fit	8.27	8	1.03	1.63	0.2844
	Pure Error	3.81	6	0.6347		
	Cor Total	84.87	17			
	Std. Dev.	0.9289				
	Mean	9.5				
	C.V. %	9.77				
	R^2	0.8575				
	Adjusted R^2	0.8371				
	Predicted R^2	0.7626				
	Adeq Precision	18.5344				
E. coli	Block	32.32	1	32.32		
	Model	1171.44	9	130.16	7.13	0.0084
	A-Temperature	32.44	1	32.44	1.78	0.2241
	B-Time	80.34	1	80.34	4.4	0.074
	AB	20.42	1	20.42	1.12	0.3252
	A^2	4.43	1	4.43	0.2427	0.6373
	B^2	22.95	1	22.95	1.26	0.299
	A^2B	19.89	1	19.89	1.09	0.3311
	AB^2	35.69	1	35.69	1.96	0.2046
	A^3	3.88	1	3.88	0.2126	0.6588
	B^3	158.01	1	158.01	8.66	0.0216
	Residual	127.7	7	18.24		
	Lack of Fit	18.81	1	18.81	1.04	0.3479
	Pure Error	108.89	6	18.15		
	Cor Total	1331.46	17			
	Std. Dev.	4.27				
	Mean	20.01				
	C.V. %	21.35				
	R^2	0.9017				
	Adjusted R^2	0.7753				
	Predicted R^2	NA[(1)]				
	Adeq Precision	11.4924				
S. aures	Block	2.59	1	2.59		
	Model	0	0			
	Residual	77.73	16	4.86		
	Lack of Fit	31.67	10	3.17	0.4125	0.8964
	Pure Error	46.06	6	7.68		
	Cor Total	80.32	17			
	Std. Dev.	2.2				
	Mean	54.03				
	C.V. %	4.08				
	R^2	0				
	Adjusted R^2	0				
	Predicted R^2	-0.203				
	Adeq Precision	1.0957				
**CT**	Block	0.1577	1	0.1577		
	Model	4.21	5	0.8416	12.14	0.0004
	A-Temperature	2.24	1	2.24	32.37	0.0001
	B-Time	0.5453	1	0.5453	7.87	0.0171
	AB	0.0361	1	0.0361	0.5203	0.4858
	A^2	2.65	1	2.65	38.28	< 0.0001
	B^2	0.0908	1	0.0908	1.31	0.2766
	Residual	0.7625	11	0.0693		
	Lack of Fit	0.5456	5	0.1091	3.02	0.1055
	Pure Error	0.2168	6	0.0361		
	Cor Total	5.13	17			
	Std. Dev.	0.2633				

	Mean	4.08				
	C.V. %	6.46				
	R^2	0.8466				
	Adjusted R^2	0.7769				
	Predicted R^2	0.1382				
	Adeq Precision	13.2735				
**CT (DP)**	Block	0.2055	1	0.2055		
	Model	0.3261	5	0.0652	3.42	0.0415
	A-Temperature	0.0442	1	0.0442	2.32	0.1559
	B-Time	0.1228	1	0.1228	6.44	0.0276
	AB	0.0323	1	0.0323	1.7	0.2193
	A^2	0.133	1	0.133	6.98	0.0229
	B^2	0.0125	1	0.0125	0.6569	0.4349
	Residual	0.2097	11	0.0191		
	Lack of Fit	0.1349	5	0.027	2.17	0.1871
	Pure Error	0.0747	6	0.0125		
	Cor Total	0.7412	17			
	Std. Dev.	0.1381				
	Mean	3.95				
	C.V. %	3.5				
	R^2	0.6086				
	Adjusted R^2	0.4308				
	Predicted R^2	-0.6825				
	Adeq Precision	6.6039				

Supplementary Table 3. Analysis of variance for the fitted models - water+chemical addition

Water + Na2CO3	Source	Sum of Squares (SS)	Degree of freedom (df)	Mean Square (MS)	*F*-value	*p*-value
**TDS**	Block	0.0029	1	0.0029		
	Model	1.61	5	0.3222	139.45	< 0.0001
	A-Temperature	0.7258	1	0.7258	314.15	< 0.0001
	B-Time	0.1805	1	0.1805	78.12	< 0.0001
	AB	0.0008	1	0.0008	0.3351	0.5743
	A^2	0.0589	1	0.0589	25.49	0.0004
	B^2	0.0433	1	0.0433	18.72	0.0012
	Residual	0.0254	11	0.0023		
	Lack of Fit	0.0096	5	0.0019	0.7286	0.6273
	Pure Error	0.0158	6	0.0026		
	Cor Total	1.64	17			
	Std. Dev.	0.0481				
	Mean	1.95				
	C.V. %	2.47				
	R^2	0.9845				
	Adjusted R^2	0.9774				
	Predicted R^2	0.9577				
	Adeq Precision	46.6111				
**FRAP**	Block	757.72	1	757.72		
	Model	259800	2	129900	20.85	< 0.0001
	A-Temperature	229400	1	229400	36.81	< 0.0001
	B-Time	37533.67	1	37533.67	6.02	0.0278
	Residual	87233.44	14	6230.96		
	Lack of Fit	85195.04	8	10649.38	31.35	0.0002
	Pure Error	2038.4	6	339.73		
	Cor Total	347800	17			
	Std. Dev.	78.94				
	Mean	826.15				
	C.V. %	9.55				
	R^2	0.7486				
	Adjusted R^2	0.7127				
	Predicted R^2	0.5083				
	Adeq Precision	12.9672				
S. aureus	Block	0.928	1	0.928		
	Model	352.67	2	176.33	5.33	0.019
	A-Temperature	349.48	1	349.48	10.57	0.0058
	B-Time	1.05	1	1.05	0.0319	0.8609
	Residual	463.09	14	33.08		
	Lack of Fit	370.75	8	46.34	3.01	0.0982
	Pure Error	92.34	6	15.39		
	Cor Total	816.69	17			
	Std. Dev.	5.75				
	Mean	23.97				
	C.V. %	23.99				
	R^2	0.4323				
	Adjusted R^2	0.3512				
	Predicted R^2	0.0329				
	Adeq Precision	6.4597				
E. coli	Block	1.76	1	1.76		
	Model	560.16	9	62.24	6.07	0.0134
	A-Temperature	0.2382	1	0.2382	0.0232	0.8832
	B-Time	23.57	1	23.57	2.3	0.1734
	AB	26.11	1	26.11	2.54	0.1547
	A^2	0.6219	1	0.6219	0.0606	0.8126
	B^2	48.64	1	48.64	4.74	0.0659
	A^2B	18.05	1	18.05	1.76	0.2264
	AB^2	0.7667	1	0.7667	0.0747	0.7925
	A^3	10.04	1	10.04	0.9785	0.3555
	B^3	88.66	1	88.66	8.64	0.0217
	Residual	71.82	7	10.26		
	Lack of Fit	19.38	1	19.38	2.22	0.187
	Pure Error	52.43	6	8.74		
	Cor Total	633.74	17			
	Std. Dev.	3.2				
	Mean	28.35				

	C.V. %	11.3				
	R^2	0.8864				
	Adjusted R^2	0.7403				
	Predicted R^2	NA[1]				
	Adeq Precision	10.2553				
**ORAC**	Block	851900	1	851900		
	Model	5599000	2	2800000	14.97	0.0003
	A-Temperature	5564000	1	5564000	29.75	< 0.0001
	B-Time	79890.5	1	79890.5	0.4272	0.524
	Residual	2618000	14	187000		
	Lack of Fit	1954000	8	244300	2.21	0.1753
	Pure Error	664300	6	110700		
	Cor Total	9069000	17			
	Std. Dev.	432.46				
	Mean	2893.93				
	C.V. %	14.94				
	R^2	0.6814				
	Adjusted R^2	0.6359				
	Predicted R^2	0.4165				
	Adeq Precision	10.8398				
**TPC**	Block	1.76	1	1.76		
	Model	635.44	4	158.86	64.48	< 0.0001
	A-Temperature	376.61	1	376.61	152.87	< 0.0001
	B-Time	10.85	1	10.85	4.4	0.0577
	AB	12.63	1	12.63	5.13	0.0429
	A^2	13.76	1	13.76	5.58	0.0358
	Residual	29.56	12	2.46		
	Lack of Fit	25.64	6	4.27	6.53	0.019
	Pure Error	3.92	6	0.6539		
	Cor Total	666.77	17			
	Std. Dev.	1.57				
	Mean	11.28				
	C.V. %	13.92				
	R^2	0.9555				
	Adjusted R^2	0.9407				
	Predicted R^2	0.8922				
	Adeq Precision	25.4719				
**CT**	Block	0.0002	1	0.0002		
	Model	0.0155	5	0.0031	17.78	< 0.0001
	A-Temperature	0.0148	1	0.0148	84.87	< 0.0001
	B-Time	0.0002	1	0.0002	1.17	0.3026
	AB	0.0002	1	0.0002	1.27	0.2838
	A^2	0.0053	1	0.0053	30.35	0.0002
	B^2	0.0002	1	0.0002	0.9837	0.3426
	Residual	0.0019	11	0.0002		
	Lack of Fit	0.0014	5	0.0003	3.15	0.0977
	Pure Error	0.0005	6	0.0001		
	Cor Total	0.0176	17			
	Std. Dev.	0.0132				
	Mean	0.0996				
	C.V. %	13.26				
	R^2	0.8899				
	Adjusted R^2	0.8399				
	Predicted R^2	0.4582				
	Adeq Precision	12.36				
**CT (DP)**	Block	0.001		1	0.001	
	Model	0.168	9	0.0187	11	0.0023
	A-Temperature	0.0546	1	0.0546	32.15	0.0008
	B-Time	0.0127	1	0.0127	7.48	0.0291
	AB	4.001E-07	1	4.001E-07	0.0002	0.9882
	A^2	0.0005	1	0.0005	0.2678	0.6207
	B^2	0.0152	1	0.0152	8.97	0.0201
	A^2B	0.0303	1	0.0303	17.87	0.0039
	AB^2	0.0167	1	0.0167	9.81	0.0166
	A^3	0.0368	1	0.0368	21.66	0.0023
	B^3	0.0017	1	0.0017	0.9927	0.3523
	Residual	0.0119	7	0.0017		

Lack of Fit	0	1	0	0.0087	0.9286
Pure Error	0.0119	6	0.002		
Cor Total	0.1809	17			
Std. Dev.	0.0412				
Mean	2				
C.V. %	2.06				
R^2	0.9339				
Adjusted R^2	0.849				
Predicted R^2	NA[(1)]				
Adeq Precision	9.31				

[(1)] Case(s) with leverage of 1.0000: Pred R^2 and PRESS statistic not defined.

Supplementary Table 4. Analysis of variance for the fitted models - limonene

Limonene	Source	Sum of Squares (SS)	Degree of freedom (df)	Mean Square (MS)	*F* -value	*p* -value
**TDS**	Block	0.0012	1	0.0012		
	Model	0.0307	3	0.0102	1.77	0.2024
	A-Temperature	0.012	1	0.012	2.08	0.1733
	B-Time	0.0147	1	0.0147	2.55	0.1345
	AB	0.0155	1	0.0155	2.67	0.126
	Residual	0.0752	13	0.0058		
	Lack of Fit	0.0371	7	0.0053	0.8335	0.5962
	Pure Error	0.0381	6	0.0064		
	Cor Total	0.1072	17			
	Std. Dev.	0.0761				
	Mean	0.84				
	C.V. %	9.06				
	R^2	0.29				
	Adjusted R^2	0.1262				
	Predicted R^2	-0.6073				
	Adeq Precision	4.8769				
**CUPRAC**	Block	16976.79	1	16976.79		
	Model	50724.23	3	16908.08	3.16	0.0608
	A-Temperature	41656.9	1	41656.9	7.79	0.0153
	B-Time	1475.77	1	1475.77	0.276	0.6082
	AB	13959.61	1	13959.61	2.61	0.1302
	Residual	69516.21	13	5347.4		
	Lack of Fit	53096.85	7	7585.26	2.77	0.1175
	Pure Error	16419.36	6	2736.56		
	Cor Total	137200	17			
	Std. Dev.	73.13				
	Mean	89.57				
	C.V. %	81.64				
	R^2	0.4219				
	Adjusted R^2	0.2884				
	Predicted R^2	-0.6567				
	Adeq Precision	6.1604				
**DPPH**	Block	0.0022	1	0.0022		
	Model	3.89	5	0.7774	9.44	0.0011
	A-Temperature	0.8002	1	0.8002	9.72	0.0098
	B-Time	0.0086	1	0.0086	0.1044	0.7527
	AB	0.0021	1	0.0021	0.0252	0.8768
	A^2	1.01	1	1.01	12.24	0.005
	B^2	0.0008	1	0.0008	0.01	0.9223
	Residual	0.906	11	0.0824		
	Lack of Fit	0.1674	5	0.0335	0.272	0.9127
	Pure Error	0.7385	6	0.1231		
	Cor Total	4.79	17			
	Std. Dev.	0.287				
	Mean	1.21				
	C.V. %	23.66				
	R^2	0.811				
	Adjusted R^2	0.7251				
	Predicted R^2	0.6796				
	Adeq Precision	10.264				
**TPC (FC)**	Block	2263.22	1	2263.22		
	Model	75049.13	5	15009.83	5.28	0.0102
	A-Temperature	9756.42	1	9756.42	3.43	0.091
	B-Time	14.05	1	14.05	0.0049	0.9452
	AB	6460.56	1	6460.56	2.27	0.1599
	A^2	26230.78	1	26230.78	9.23	0.0113
	B^2	7403.63	1	7403.63	2.6	0.1349
	Residual	31274.18	11	2843.11		

Lack of Fit	11626.82	5	2325.36	0.7101	0.6379
Pure Error	19647.36	6	3274.56		
Cor Total	108600	17			
Std. Dev.	53.32				
Mean	105.55				
C.V. %	50.52				
R^2	0.7059				
Adjusted R^2	0.5722				
Predicted R^2	0.2427				
Adeq Precision	8.2414				

Supporting Information - Supplementary table 5: Individual responses for optimization

Antioxidant activity (FRAP, ORAC), total phenolic content (TPC), and antibacterial properties (*E. coli* and *S. aureus*)

		FRAP (µM Fe(II) eq. /g)				ORAC (µM TE/g)				TPC (mg GAE/g)				*E. coli* (% inhibition)				*S. aureus* (% inhibition)			
**Ethanol**	Factors	average	st dev	cv (%)	median	average	st dev	cv (%)	median	average	st dev	cv (%)	median	average	st dev	cv (%)	median	average	st dev	cv (%)	median
1	120 °C, 60min	410.8	14.8	3.6	404.9	2800.5	275.4	9.8	2819.0	14.1	1.0	6.9	14.2	39.2	1.4	3.6	38.6	54.7	2.8	5.1	54.2
2	85 °C, 35min	332.7	6.0	1.8	333.1	2158.7	194.2	9.0	2175.8	11.5	0.6	5.1	11.6	24.4	2.3	9.4	25.3	52.3	0.4	0.8	54.1
3	50 °C, 60min	239.2	12.8	5.3	243.8	1660.9	154.4	9.3	1642.0	7.0	0.4	6.4	7.2	21.4	1.9	8.8	20.4	52.6	0.7	1.3	54.2
4	85 °C, 35min	319.3	4.6	1.4	319.9	2142.0	168.5	7.9	2118.3	10.4	0.1	1.4	10.4	23.2	1.2	5.0	23.5	48.7	2.1	4.4	54.2
5	50 °C, 10min	174.2	4.5	2.6	172.9	1121.4	100.8	9.0	1114.0	4.6	0.4	8.2	4.6	0.9	0.8	84.9	0.9	53.3	0.8	1.6	54.2
6	85 °C, 70min	369.4	12.6	3.4	372.7	2207.1	166.4	7.5	2220.5	11.1	0.1	1.0	11.1	17.1	0.9	5.4	17.2	55.8	1.4	2.6	54.2
7	40 °C, 35min	173.0	7.1	4.1	172.4	1238.4	73.8	6.0	1222.0	5.3	0.1	2.1	5.3	8.4	0.2	2.1	8.4	53.8	1.3	2.3	53.9
8	135 °C, 35min	382.7	12.3	3.2	385.1	2356.2	210.8	8.9	2319.8	11.5	0.8	6.7	11.1	20.4	1.8	9.0	20.5	57.5	2.0	3.6	53.8
9	85 °C, 35min	347.5	3.2	0.9	346.9	2053.9	173.9	8.5	2074.3	9.2	0.3	2.9	9.0	20.9	4.6	22.2	20.9	57.6	0.5	0.8	53.2
10	120 °C, 10min	339.3	8.8	2.6	343.0	3009.8	273.8	9.1	2976.8	10.6	0.5	4.5	10.7	34.4	2.7	7.7	34.4	55.1	0.5	1.0	51.9
11	85 °C, 35min	350.0	7.1	2.0	352.6	1676.7	75.0	4.5	1679.1	9.4	0.4	4.0	9.6	16.3	3.0	18.1	16.3	53.0	1.3	2.4	35.2
12	85 °C, 35min	327.5	3.6	1.1	327.4	1785.8	178.1	10.0	1802.0	9.8	0.4	4.3	9.8	24.8	1.3	5.2	24.8	50.8	1.3	2.6	19.0
13	103 °C, 48min	372.5	4.7	1.3	370.0	2037.1	166.5	8.2	2073.4	10.4	0.2	1.6	10.3	26.6	0.5	2.0	26.6	53.4	1.4	2.6	17.6
14	85 °C, 35min	334.4	4.0	1.2	334.9	1761.4	146.0	8.3	1776.9	8.8	0.5	5.2	8.9	19.9	1.9	9.4	20.6	56.0	1.5	2.7	15.7
15	85 °C, 35min	336.0	9.9	2.9	340.5	1758.3	159.9	9.1	1731.0	9.5	0.2	2.3	9.5	21.2	0.9	4.4	21.2	54.2	1.5	2.7	14.2
16	68 °C, 48min	271.3	0.8	0.3	271.6	1504.7	123.5	8.2	1516.2	8.4	0.3	3.9	8.2	16.2	0.9	5.7	16.2	54.9	3.2	5.8	13.7
17	103 °C, 23min	333.5	6.9	2.1	336.3	1824.1	170.6	9.4	1780.1	10.1	0.2	2.1	10.0	13.8	0.7	5.3	14.0	54.7	1.8	3.3	13.6
18	85 °C, 35min	339.5	3.1	0.9	340.8	1719.1	144.5	8.4	1652.4	9.3	0.5	5.0	9.6	11.1	0.8	7.5	11.0	54.1	0.6	1.0	13.5
max		410.8				3009.8				14.1				39.2				57.6			
min		173.0				1121.4				4.6				0.9				48.7			
		FRAP (µM Fe(II) eq. /g)				ORAC (µM TE/g)				TPC (mg GAE/g)				*E. coli* (% inhibition)				*S. aureus* (% inhibition)			
**H2O**	Factors	average	st dev	cv (%)	median	average	st dev	cv (%)	median	average	st dev	cv (%)	median	average	st dev	cv (%)	median	average	st dev	cv (%)	median
1	120 °C, 60min	355.1	4.6	1.3	355.3	1766.4	166.5	9.4	1821.1	11.8	0.3	2.4	11.9	11.1	0.5	4.3	11.1	18.5	0.2	0.9	13.2
2	85 °C, 35min	271.9	12.7	4.7	274.7	1594.5	107.9	6.8	1629.8	9.8	0.7	7.3	9.8	4.9	0.1	2.6	4.9	13.1	0.4	2.9	13.1
3	40 °C, 35min	169.6	1.4	0.8	169.5	841.0	49.6	5.9	839.4	4.9	0.3	6.5	4.8	3.6	0.3	8.6	3.6	9.7	0.0	0.0	13.2
4	50 °C, 60min	197.2	3.0	1.5	197.1	1053.9	84.4	8.0	1043.3	4.8	0.0	0.9	4.8	3.4	0.1	2.2	3.4	10.8	0.2	1.4	13.5
5	85 °C, 35min	262.2	10.2	3.9	262.5	1567.0	131.3	8.4	1594.4	8.3	0.4	5.3	8.3	6.1	0.2	2.8	6.1	14.7	0.7	4.6	13.6
6	50 °C, 10min	141.6	2.6	1.8	143.1	728.5	27.7	3.8	718.7	4.3	0.3	7.8	4.3	4.5	0.1	1.2	4.5	9.6	0.3	3.0	13.6
7	85 °C, 70min	257.8	5.0	2.0	257.2	1510.9	102.0	6.8	1467.5	10.1	0.8	7.7	10.0	4.6	0.1	1.6	4.6	13.4	0.4	2.8	13.7
8	135 °C, 35min	340.8	23.1	6.8	345.8	1849.1	128.8	7.0	1835.9	12.7	1.2	9.1	12.5	10.6	0.1	1.0	10.6	19.5	0.3	1.4	13.8
9	85 °C, 35min	243.8	10.7	4.4	249.8	1483.4	127.2	8.6	1537.7	8.7	0.8	9.5	8.3	5.0	0.3	5.9	5.0	13.8	0.6	4.0	13.8
10	120 °C, 10min	299.3	14.3	4.8	301.9	2126.0	160.6	7.6	2179.3	11.2	0.7	6.5	11.2	11.1	0.5	4.3	11.1	16.7	0.0	0.2	14.6
11	85 °C, 35min	251.8	12.8	5.1	250.9	1537.2	62.4	4.1	1559.8	9.4	0.8	8.1	9.6	4.9	0.1	2.6	4.9	10.8	0.8	7.7	14.6
12	85 °C, 35min	254.4	13.8	5.4	255.5	1656.3	134.8	8.1	1651.4	9.3	0.8	8.3	9.3	3.6	0.3	8.6	3.6	11.4	0.1	0.9	17.1
13	103 °C, 48min	328.9	18.2	5.5	337.8	1908.8	163.8	8.6	1968.9	12.9	0.8	6.1	12.9	3.4	0.1	2.2	3.4	13.6	0.3	2.1	19.5
14	85 °C, 35min	254.3	11.4	4.5	260.0	1561.4	117.1	7.5	1547.2	11.0	0.5	4.8	11.0	6.1	0.2	2.8	6.1	13.6	0.1	1.0	19.5
15	85 °C, 35min	270.1	14.6	5.4	269.9	1919.8	127.3	6.6	1878.9	8.9	0.4	4.9	8.7	4.5	0.1	1.2	4.5	12.0	0.3	2.5	19.5
16	68 °C, 48min	220.0	11.3	5.1	217.3	1182.8	55.9	4.7	1186.4	9.1	0.7	8.1	9.3	4.6	0.1	1.6	4.6	10.2	0.6	6.1	20.8
17	103 °C, 23min	259.5	11.5	4.4	256.0	1479.4	78.5	5.3	1516.4	7.8	0.3	3.2	7.9	10.6	0.1	1.0	10.6	13.7	0.5	3.9	21.9
18	85 °C, 35min	258.6	20.8	8.0	266.8	1458.0	117.9	8.1	1485.0	6.5	0.6	9.9	6.2	5.0	0.3	5.9	5.0	13.1	1.0	7.3	23.0
max		355.1				2126.0				12.9				11.1				19.5			
min		141.6				728.5				4.3				3.4				9.6			
		FRAP (µM Fe(II) eq. /g)				ORAC (µM TE/g)				TPC (mg GAE/g)				*E. coli* (% inhibition)				*S. aureus* (% inhibition)			
**H2O+Na2SO3+Na2CO3**	Factors	average	st dev	cv (%)	median	average	st dev	cv (%)	median	average	st dev	cv (%)	median	average	st dev	cv (%)	median	average	st dev	cv (%)	median
1	120 °C, 60min	1045.7	17.6	1.7	1045.6	4388.2	151.3	3.4	4405.2	23.8	1.1	4.8	23.3	37.0	2.0	5.3	36.2	28.0	1.9	6.8	23.0
2	85 °C, 35min	762.2	30.1	4.0	771.5	2723.5	264.9	9.7	2732.7	10.1	0.5	4.6	10.3	23.8	0.8	3.3	23.8	18.7	0.5	2.8	22.5
3	40 °C, 35min	671.5	16.9	2.5	667.3	1753.1	67.2	3.8	1747.5	2.4	0.0	0.7	2.4	23.6	0.4	1.8	23.6	15.5	0.1	0.5	23.0
4	50 °C, 60min	670.1	23.2	3.5	666.7	2151.2	207.9	9.7	2127.5	3.6	0.3	9.0	3.7	31.6	0.0	0.1	31.6	20.3	0.3	1.5	23.6
5	85 °C, 35min	758.9	17.6	2.3	756.6	2947.2	291.4	9.9	3017.8	9.0	0.9	9.8	8.9	28.5	0.0	0.0	28.5	21.3	0.3	1.5	23.8
6	50 °C, 10min	659.4	16.9	2.6	659.4	2712.2	180.1	6.6	2619.1	3.0	0.3	8.8	2.9	20.3	0.9	4.5	20.7	22.5	1.8	7.8	24.1
7	85 °C, 70min	1059.3	39.2	3.7	1078.4	3005.8	262.3	8.7	2987.4	12.1	0.4	3.6	11.9	15.2	0.6	4.0	15.2	24.1	1.4	5.7	24.2
8	135 °C, 35min	1114.1	37.8	3.4	1109.8	3835.5	258.5	6.7	3950.9	22.8	0.5	2.0	23.0	40.9	0.5	1.3	40.9	43.5	0.2	0.5	24.2
9	85 °C, 35min	778.7	39.7	5.1	769.4	2684.6	249.2	9.3	2601.3	8.8	0.6	6.6	9.1	28.8	0.4	1.3	28.8	26.2	0.7	2.5	23.9
10	120 °C, 10min	836.0	12.7	1.5	831.3	3816.8	158.8	4.2	3860.7	16.5	0.9	5.5	16.1	32.4	0.6	1.7	32.4	30.8	0.3	1.1	23.6
11	85 °C, 35min	739.9	11.2	1.5	738.5	3434.8	226.7	6.6	3536.8	10.9	0.6	5.4	10.9	31.5	1.6	5.1	31.5	16.5	1.0	6.3	21.1
12	85 °C, 35min	763.2	11.9	1.6	763.2	3120.3	185.1	5.9	3110.4	9.6	0.7	6.9	9.3	29.3	0.8	2.6	29.0	18.3	0.7	4.0	23.6
13	103 °C, 48min	927.5	38.7	4.2	925.1	3785.4	217.0	5.7	3807.2	18.9	1.2	6.5	18.4	31.1	0.6	1.9	31.4	24.2	1.2	4.8	23.9
14	85 °C, 35min	783.9	34.6	4.4	795.0	2669.4	186.1	7.0	2696.8	8.6	0.4	4.7	8.6	24.9	0.3	1.3	24.9	31.5	0.7	2.4	23.6
15	85 °C, 35min	825.7	17.2	2.1	817.3	2773.4	174.9	6.3	2838.5	10.1	0.9	8.9	10.2	22.2	0.7	3.3	22.1	23.6	1.7	7.2	21.1
16	68 °C, 48min	691.0	24.4	3.5	695.5	2029.3	156.7	7.7	2077.9	6.2	0.4	6.3	6.2	33.2	0.7	2.0	33.1	18.5	0.2	0.9	18.5
17	103 °C, 23min	1003.5	12.8	1.3	1000.9	2184.8	137.6	6.3	2133.5	17.0	1.0	5.6	16.5	27.4	1.3	4.7	26.7	18.0	0.8	4.4	24.0
18	85 °C, 35min	780.3	21.2	2.7	780.3	2075.4	137.8	6.6	2139.0	9.5	0.7	7.5	9.6	28.6	0.4	1.4	28.6	29.9	1.7	5.7	29.9
max		1114.1				4388.2				23.8				40.9				43.5			
min		659.4				1753.1				2.4				15.2				15.5			

		CUPRAC (mg AAE/g)				DPPH (mg AAE/g)				TPC (mg GAE/g)			
**Limonene**	Factors	average	stdev	CV (%)	median	average	stdev	CV (%)	median	average	stdev	CV (%)	median
55	120 °C; 60min	315.6	12.2	3.9	315.6	1.8	0.1	3.2	1.8	277.0	2.7	1.0	277.0
56	85 °C; 35min	34.8	1.0	2.9	34.6	0.6	0.0	0.0	0.6	64.6	1.3	2.0	64.6
57	47 °C; 35min	42.7	2.1	4.9	41.5	0.8	0.1	8.9	0.8	38.6	0.0	0.0	38.6
58	85 °C; 35min	30.4	1.2	3.8	30.6	0.6	0.1	13.7	0.6	54.5	2.9	5.3	54.5
59	50 °C; 10min	24.6	0.4	1.5	24.4	0.7	0.1	10.4	0.7	61.6	0.0	0.0	61.6
60	85 °C; 70min	14.5	1.0	6.7	14.0	1.0	0.0	3.4	1.0	16.7	1.3	7.6	16.7
61	36 °C; 35min	28.9	0.5	1.6	28.9	1.0	0.1	6.9	0.9	113.8	1.3	1.1	113.8
62	135 °C; 35min	59.2	12.4	20.9	54.5	2.6	0.5	18.9	2.3	296.5	2.1	0.7	296.5
63	85 °C; 35min	28.3	1.5	5.2	27.4	1.0	0.0	3.3	1.0	179.8	4.0	2.2	182.1
64	120 °C; 10min	108.8	2.5	2.3	108.8	1.9	0.2	8.9	1.9	139.6	1.7	1.2	139.6
65	85 °C; 35min	60.3	1.8	2.9	60.3	1.3	0.2	12.3	1.3	109.6	0.0	0.0	109.6
66	85 °C; 35min	66.2	4.7	7.1	69.0	1.2	0.1	10.3	1.2	9.6	0.9	9.0	9.6
67	103 °C; 48min	270.7	5.6	2.1	270.7	1.9	0.2	9.2	1.9	94.1	4.1	4.3	94.1
68	85 °C; 35min	165.3	14.3	8.7	165.3	1.4	0.2	13.3	1.4	96.0	5.5	5.7	96.0
69	85 °C; 35min	187.9	12.0	6.4	187.9	1.1	0.2	20.4	1.2	77.8	2.1	2.7	79.0
70	68 °C; 48min	24.4	0.9	3.6	23.8	1.0	0.2	19.0	0.9	76.6	2.9	3.8	76.6
71	103 °C; 23min	122.6	0.0	0.0	122.6	1.5	0.2	15.0	1.5	41.1	1.9	4.6	40.0
72	85 °C; 35min	27.1	0.4	1.5	27.4	0.6	0.2	26.7	0.6	152.6	0.0	0.0	152.6

Condensed tannins

**Ethanol**	Factors	Condensed tannins (g/100g)	Degree of polymerization, DP	PC (%)	PD (%)
1	120 °C, 60min	4.7 ± 0.1	3.62 ± 0.06	95.8	4.2
2	85 °C, 35min	4.5 ± 0.1	3.94 ± 0.01	96.8	3.2
3	50 °C, 60min	4.0 ± 0.02	3.61 ± 0.07	95.9	4.1
4	85 °C, 35min	4.6 ± 0.2	3.84 ± 0.09	96.4	3.6
5	50 °C, 10min	3.1 ± 0.02	3.79 ± 0.04	96.4	3.6
6	85 °C, 70min	4.6 ± 0.1	3.68 ± 0.01	95.4	4.6
7	40 °C, 35min	2.7 ± 0.01	3.77 ± 0.01	96.5	3.5
8	135 °C, 35min	3.1 ± 0.01	3.69 ± 0.06	97.2	2.8
9	85 °C, 35min	4.1 ± 0.02	4.17 ± 0.02	97.3	2.7
10	120 °C, 10min	4.1 ± 0.1	4.22 ± 0.02	97.5	2.5
11	85 °C, 35min	4.4 ± 0.05	4.03 ± 0.04	97.2	2.8
12	85 °C, 35min	4.2 ± 0.1	4.08 ± 0.07	97.2	2.8
13	103 °C, 48min	4.5 ± 0.1	4.17 ± 0.01	97.3	2.7
14	85 °C, 35min	4.3 ± 0.03	4.18 ± 0.05	97.6	2.4
15	85 °C, 35min	4.1 ± 0.03	4.04 ± 0.01	97.0	3.0
16	68 °C, 48min	4.0 ± 0.1	4.13 ± 0.04	97.2	2.8
17	103 °C, 23min	4.3 ± 0.1	3.97 ± 0.03	97.2	2.8
18	85 °C, 35min	4.1 ± 0.1	4.09 ± 0.02	97.7	2.3
**H2O**	Factors	Condensed tannins (g/100g)	Degree of polymerization, DP	PC (%)	PD (%)
1	120 °C, 60min	3.3 ± 0.1	3.31 ± 0.01	96.8	3.2
2	85 °C, 35min	4.9 ± 0.07	3.47 ± 0.10	96.3	3.7
3	40 °C, 35min	2.9 ± 0.01	3.37 ± 0.06	97.0	3.0
4	50 °C, 60min	3.3 ± 0.03	3.34 ± 0.01	97.2	2.8
5	85 °C, 35min	4.6 ± 0.1	3.57 ± 0.01	96.9	3.1
6	50 °C, 10min	3.1 ± 0.1	3.44 ± 0.05	97.0	3.0
7	85 °C, 70min	4.4 ± 0.04	3.57 ± 0.05	97.0	3.0
8	135 °C, 35min	2.3 ± 0.05	3.01 ± 0.03	96.9	3.1
9	85 °C, 35min	4.4 ± 0.01	3.64 ± 0.02	96.9	3.1
10	120 °C, 10min	4.8 ± 0.2	3.84 ± 0.08	97.0	3.0
11	85 °C, 35min	4.3 ± 0.1	3.63 ± 0.05	96.9	3.1
12	85 °C, 35min	4.3 ± 0.1	3.58 ± 0.03	96.7	3.3
13	103 °C, 48min	4.5 ± 0.1	3.73 ± 0.01	97.1	2.9
14	85 °C, 35min	4.2 ± 0.1	3.70 ± 0.04	97.5	2.5
15	85 °C, 35min	4.2 ± 0.03	3.63 ± 0.04	97.1	2.9
16	68 °C, 48min	3.5 ± 0.1	3.41 ± 0.05	96.8	3.2
17	103 °C, 23min	4.3 ± 0.01	3.88 ± 0.06	96.9	3.1
18	85 °C, 35min	4.1 ± 0.03	3.54 ± 0.03	97.1	2.9
**H2O+Na2SO3+N**	Factors	Condensed tannins (g/100g)	Degree of polymerization, DP	PC (%)	PD (%)
1	120 °C, 60min	0.089 ± 0.005	1.86 ± 0.07	100	ND
2	85 °C, 35min	0.076 ± 0.002	2.11 ± 0.02	100	ND
3	40 °C, 35min	0.161 ± 0.005	1.89 ± 0.01	100	ND
4	50 °C, 60min	0.172 ± 0.003	1.87 ± 0.01	100	ND
5	85 °C, 35min	0.078 ± 0.002	2.04 ± 0.03	100	ND
6	50 °C, 10min	0.148 ± 0.010	1.88 ± 0.02	100	ND
7	85 °C, 70min	0.076 ± 0.003	2.10 ± 0.05	100	ND
8	135 °C, 35min	0.084 ± 0.001	1.85 ± 0.03	100	ND
9	85 °C, 35min	0.085 ± 0.002	2.12 ± 0.02	100	ND
10	120 °C, 10min	0.089 ± 0.007	2.01 ± 0.08	100	ND
11	85 °C, 35min	0.078 ± 0.006	2.09 ± 0.03	100	ND
12	85 °C, 35min	0.085 ± 0.001	2.09 ± 0.05	100	ND
13	103 °C, 48min	0.077 ± 0.005	2.15 ± 0.11	100	ND
14	85 °C, 35min	0.077 ± 0.003	2.03 ± 0.08	100	ND
15	85 °C, 35min	0.089 ± 0.002	2.03 ± 0.06	100	ND
16	68 °C, 48min	0.141 ± 0.003	1.90 ± 0.03	100	ND
17	103 °C, 23min	0.080 ± 0.001	2.02 ± 0.07	100	ND
18	85 °C, 35min	0.107 ± 0.002	1.92 ± 0.03	100	ND

Supplementary Table 6: The theoretical and experimental results of ethanol extraction at 110 °C and 60 min.

	**Predicted Mean**	**95% Tolerance level for 99% Population**		**Observed Mean**	**Stdev**
		**low**	**high**		
**TDS** (Wt%)	3.36	2.99	3.74	3.55	0.00
**TDS** (mg/g)	230.08	205.35	254.81	247.74	0.16
**FRAP**, μM Fe(II) eq./g	399.25	338.71	459.78	504.75	32.19
**ORAC**, μM TE/g	2376.73	1266.86	3486.60	2740.30	213.93
**TPC**, mg GAE/g	12.48	8.16	16.79	13.22	0.20
E. coli , inh%	33.47	7.19	59.74	10.64	2.87
S. aureus , inh%	54.16	45.13	63.19	43.93	1.14

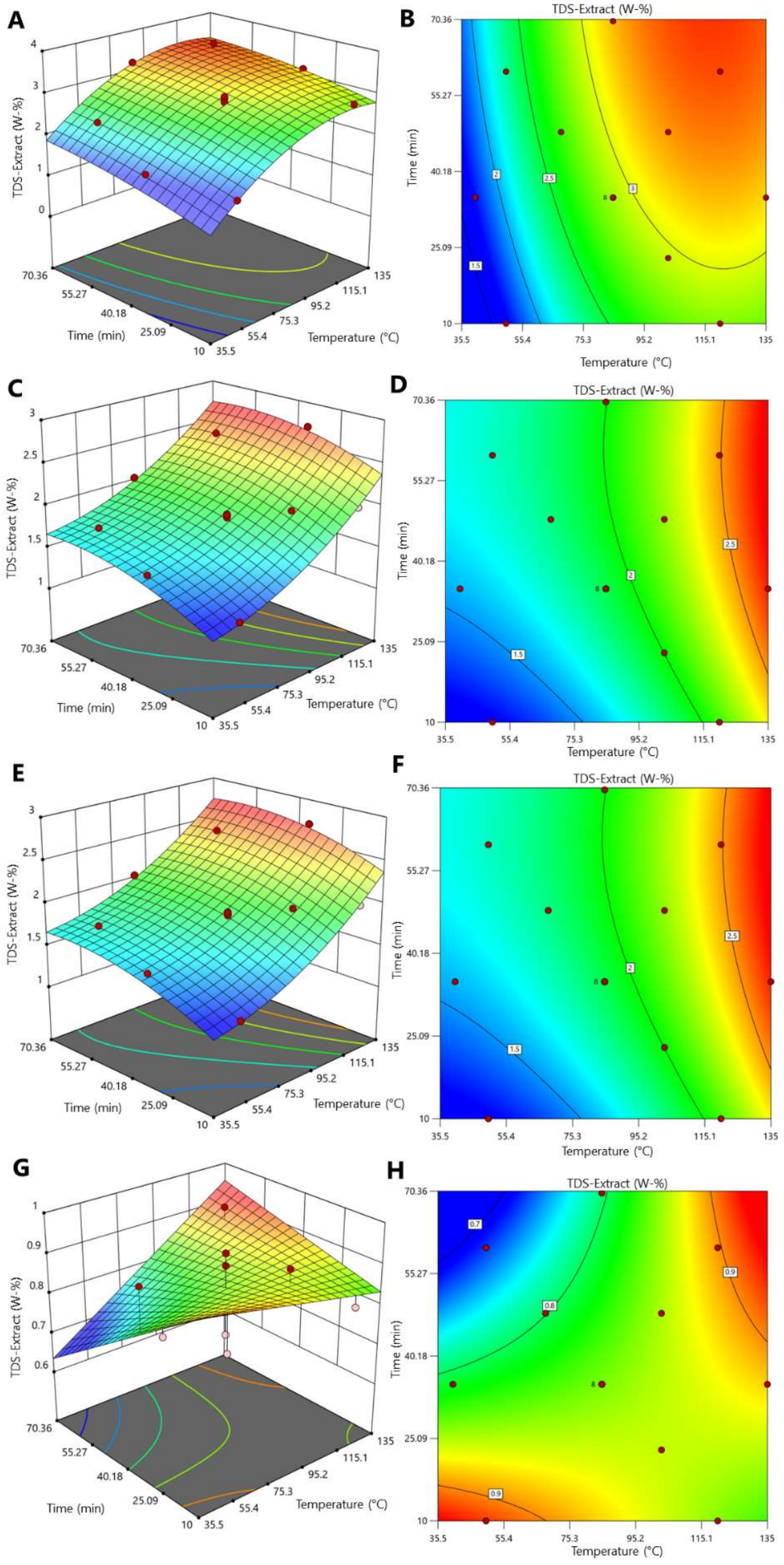

A
TDS-Extract (W-%)
Time (min)
Temperature (°C)
B
TDS-Extract (W-%)
Time (min)
Temperature (°C)
C
TDS-Extract (W-%)
Time (min)
Temperature (°C)
D
TDS-Extract (W-%)
Time (min)
Temperature (°C)
E
TDS-Extract (W-%)
Time (min)
Temperature (°C)
F
TDS-Extract (W-%)
Time (min)
Temperature (°C)
G
TDS-Extract (W-%)
Time (min)
Temperature (°C)
H
TDS-Extract (W-%)
Time (min)
Temperature (°C)

Supplementary Figure 1. Total dissolved solids (TDS) per extract weight-% RSM is quadratic for aq. ethanol (R^2=0.9754) (3D surface A, contour plot B), water (R^2=0.9774) (3D surface C, contour plot D), water + Na_2CO_3 and $NaHSO_3$ (R^2=0.9774) (3D surface E, contour plot F) and two-fraction interaction (2FI) for limonene (R^2=0.1262) (3D surface G, contour plot H) extract.

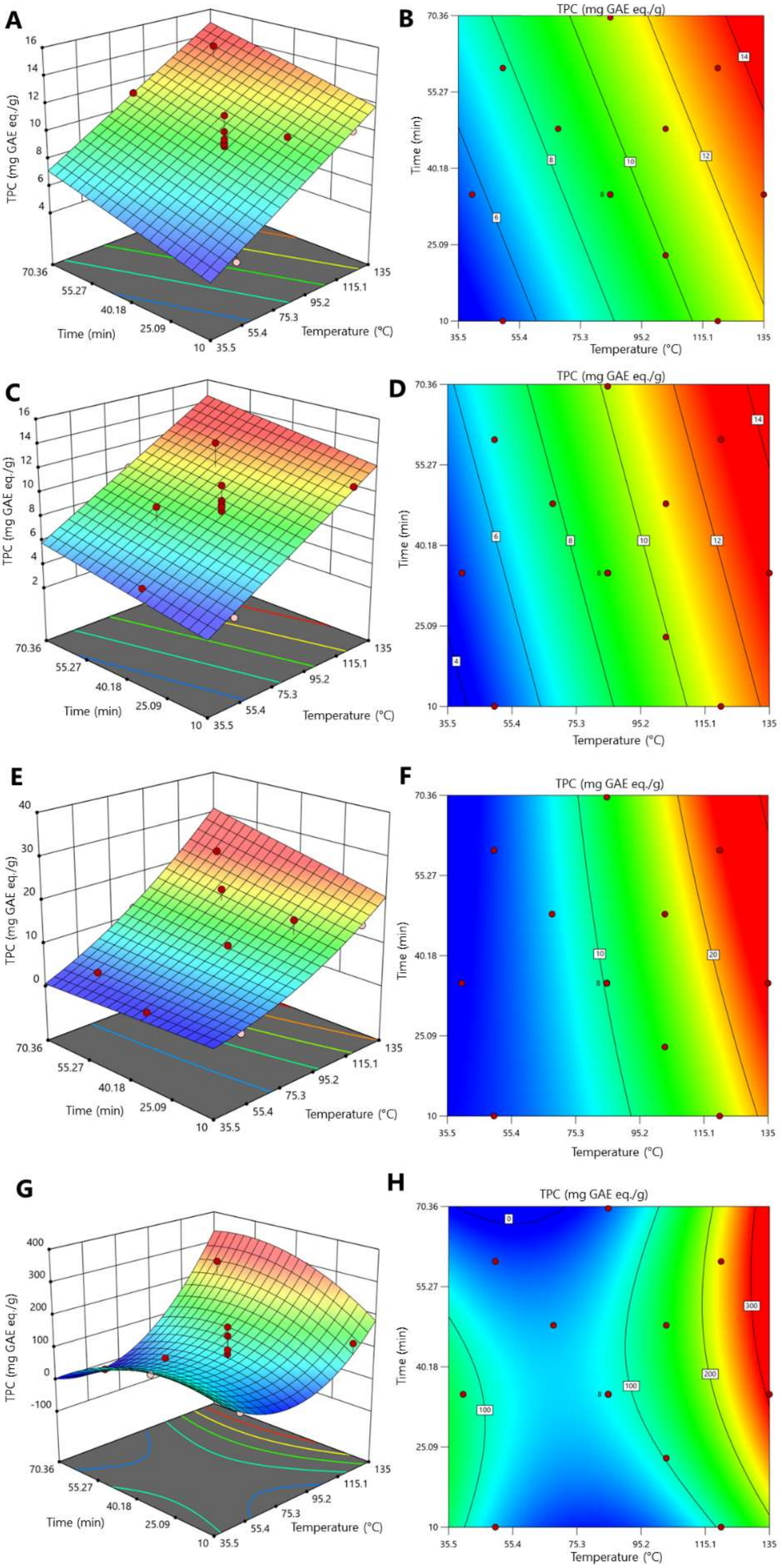

A
B
C
D
E
F
G
H
TPC (mg GAE eq./g)
Time (min)
Temperature (°C)
70.36
55.27
40.18
25.09
10
35.5
55.4
75.3
95.2
115.1
135

Supplementary Figure 2. Total phenolic content (TPC) in samples (mg GAE/g). Prussian blue method was used for aq. ethanol (3D surface in A and contour plot in B), water (3D surface in C and contour plot in D), water + Na_2CO_3 and $NaHSO_3$ (3D surface in E and contour plot in F) and modified Folin-Ciocalteu method was used for limonene (3D surface in G and contour plot in H) extracts. Response surface model was linear for aq. ethanol (R^2=0.8371), linear for water (R^2=0.7262), modified quadratic (without time^2 term) for water + Na_2CO_3 and $NaHSO_3$ (R^2=0.9386), and quadratic for limonene (R^2=0.5722).

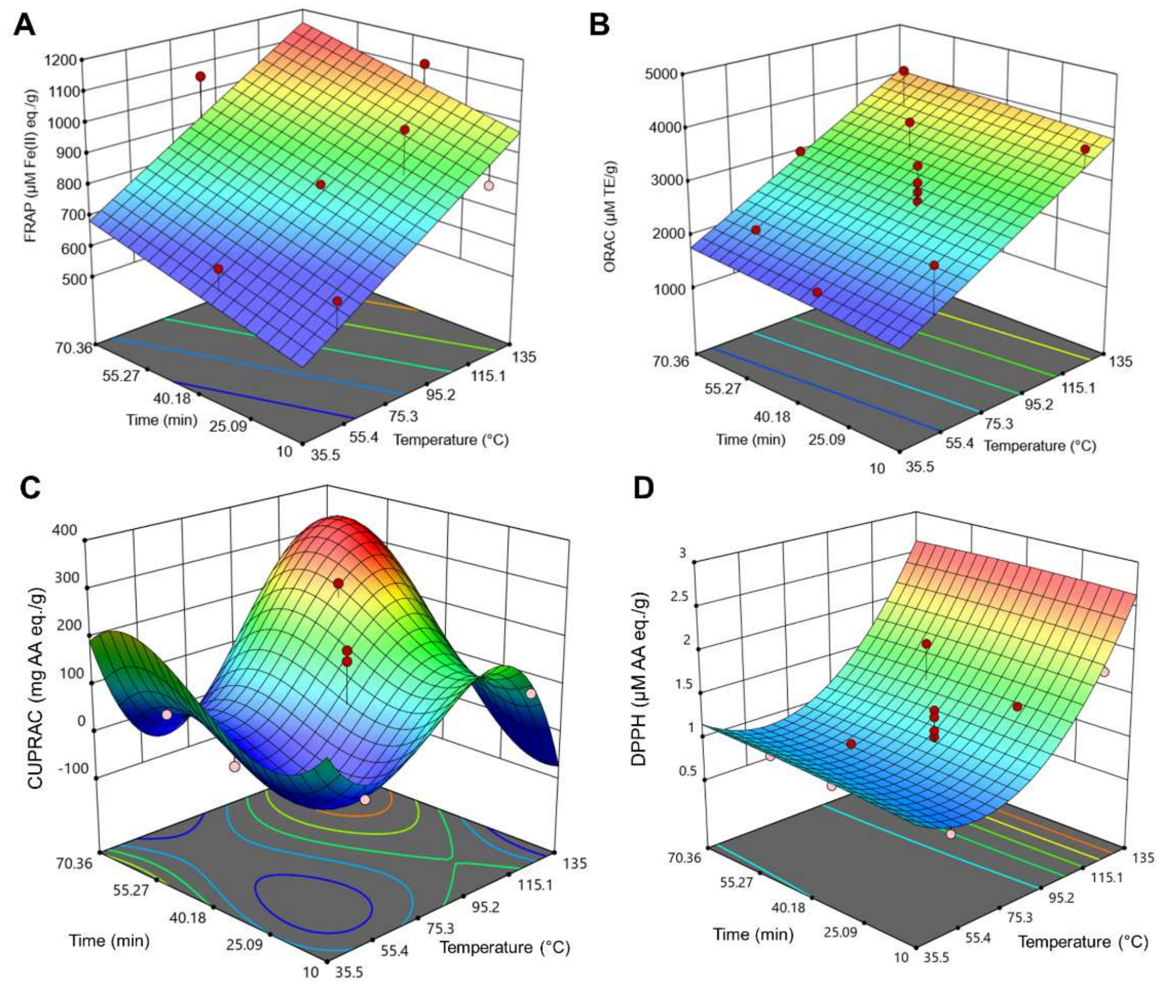

Supplementary Figure 3. Antioxidant activity test results for water + Na_2CO_3 and $NaHSO_3$ extracts (A) FRAP (μM Fe(II) eq./g) and (B) ORAC (μM TE/g) and limonene extracts (C) CUPRAC (mg AA eq./g) and (D) DPPH (μM AA eq./g). Response surface model was linear for A (R^2=0.7127) and B (R^2=0.6359), cubic for C (R^2=0.6009), and quadratic for D (R^2=0.7251).
